# A computational model of feedback-mediated hematopoietic stem cell differentiation *in vitro*

**DOI:** 10.1371/journal.pone.0212502

**Published:** 2019-03-01

**Authors:** Bhushan Mahadik, Bruce Hannon, Brendan A. C. Harley

**Affiliations:** 1 Carl R. Woese Institute for Genomic Biology, University of Illinois at Urbana-Champaign, Urbana, Illinois, United States of America; 2 Dept. of Chemical and Biomolecular Engineering, University of Illinois at Urbana-Champaign, Urbana, Illinois, United States of America; 3 Liberal Arts and Sciences, University of Illinois at Urbana-Champaign, Urbana, Illinois, United States of America; Wake Forest Institute for Regenerative Medicine, UNITED STATES

## Abstract

Hematopoietic stem cells (HSCs) play an important physiological role as regulators of all blood and immune cell populations, and are of clinical importance for bone marrow transplants. Regulating HSC biology in vitro for clinical applications requires improved understanding of biological inducers of HSC lineage specification. A significant challenge for controlled HSC expansion and differentiation is the complex network of molecular crosstalk between multiple bone marrow niche components influencing HSC biology. We describe a biology-driven computational approach to model cell kinetics in vitro to gain new insight regarding culture conditions and intercellular signaling networks. We further investigate the balance between self-renewal and differentiation that drives early and late hematopoietic progenitor populations. We demonstrate that changing the feedback driven by cell-secreted biomolecules alters lineage specification in early progenitor populations. Using a first order deterministic model, we are able to predict the impact of media change frequency on cell kinetics, as well as distinctions between primitive long-term HSCs and differentiated myeloid progenitors. Integrating the computational model and sensitivity analyses we identify critical culture parameters for regulating HSC proliferation and myeloid lineage specification. Our analysis suggests that accurately modeling the kinetics of hematopoietic sub-populations in vitro requires direct contributions from early progenitor differentiation along with the more traditionally considered intermediary oligopotent progenitors. While consistent with recent in vivo results, this work suggests the need to revise our perspective on HSC lineage engineering in vitro for expansion of discrete hematopoietic populations.

## Introduction

Hematopoietic stem cells (HSC) mediate production of the body’s full complement of blood and immune cells through a series of linked differentiation steps. HSCs are primarily located in distinct niche microenvironments in the bone marrow where their activity is strongly influenced by a constellation of molecular signals from local niche components including other niche cells, extracellular matrix (ECM), and soluble or ECM-immobilized biomolecular signals[1, 2]. Primary stem cell fates associated with hematopoietic homeostasis such as differentiation, proliferation, and quiescence are regulated by these signaling mechanisms, making the hematopoietic system highly responsive to external stimuli[3–6]. Dysregulation within the HSC niche can also play a significant role in the onset of a range of hematopoietic pathologies such as cancers (leukemia, lymphoma, myelodysplasia) and degenerative conditions (bone marrow failure) [7, 8].

Hematopoietic stem cell transplantation is an important element of successful therapies for a range of hematopoietic pathologies that require myelo-ablative treatments. Here, the immuno-compromised recipient bone marrow is fully reconstituted by transplantation of a small fraction of healthy and highly repopulating CD34^+^ HSCs from a donor [9]. The success or failure of this process can be strongly dependent on the number of functional long-term repopulating HSCs and often low numbers lead to poor engraftment, reduced platelet and neutrophil recovery and negatively impact patient survival [10, 11]. Efforts are aimed at *in vitro* expansion of functional HSCs in liquid cultures via a cocktail of cytokines and small molecules prior to transplantation, which offer an opportunity of improved engraftment either through expansion of donor HSCs or preconditioning them towards improved homing and engraftment in the recipient. However, such efforts face the challenge of uncontrolled HSC differentiation and the loss of the functional long-term repopulating stem cells *in vitro*[12]. Indeed, an optimal culture environment for targeted hematopoietic expansion may need to emulate aspects of the native niche interactions between HSCs and key cellular, matrix and biomolecular niche components that trigger the necessary HSC response. While much is known about the underlying signaling mechanisms governing these interactions, replicating them *in vitro* to successfully guide HSC fate remains challenging. One significant element of this challenge is the need to develop a framework that explicitly defines and examines the effects of complex *in vitro* cultures.

Computational approaches may provide a critical resource for engineering an optimal HSC culture environment, specifically via its capacity to identify key niche components that drive the vital balance between stem cell self-renewal vs. differentiation *in vitro*. Such efforts also offer the opportunity to significantly reduce the number of expensive, time-consuming experiments that need to be performed. This is particularly important in the context of scaling up culture systems for HSC biomanufacturing to address the clinical need for donor HSCs. Previous efforts have investigated the application of mathematical models to study the impact of signaling networks on cell fate determination. In particular, the importance of cytokine-mediated feedback mechanisms in determining lineage specification for stem cells and cancer progression is well established [13–15]. *Stiehl et al*. have adapted mathematical modeling approaches to correlate cytokine response of leukemia patients suffering with treatment response and overall survival[16] as well as predict acute myeloid leukemia survival from HSC frequencies in patients[17]. Experimental investigations of HSC lineage specification processes have established the importance of dynamically regulating feedback-mediated cell-cell signaling, cell-secreted biomolecules,[18–21] and exogenously added cytokines[12] to alter the kinetics of stem cell lineage specification and progenitor populations. A common theme across these studies is the positive and negative effects as well as feedback loops associated with cell-secreted cytokines that dynamically alters stem cell behavior.

Here we examine the balance between stem cell self-renewal vs. differentiation and proliferation at the apex of the HSC hierarchy using a combination of biology and computational methods. Our efforts concentrate on demonstrating a means to develop a modeling framework to facilitate studying feedback from cell-secreted biomolecules in the context of the differentiation of early and late progenitors into mature hematopoietic populations. We explore the use of parameter sensitivity analyses to identify principal instructive elements as well as the predictive nature of the model to describe HSC response to perturbations in culture conditions. These studies bring us closer to creating a holistic model of an engineered, highly regulated HSC culture system capable of targeted hematopoietic cell expansion for clinical purposes.

## Materials and methods

All work involving primary cells harvested from animals (mouse) was approved and conducted under approached animal welfare protocols by Institutional Animal Care and Use Committee (IACUC), University of Illinois at Urbana-Champaign, Animal Use Protocol #14202. All animals were housed under standard IACUC procedures. Animals were euthanized with CO2 under proper IACUC guidelines prior to tissue dissection for harvesting the primary stem cells.

### HSC isolation

Primary HSCs were isolated from the bone marrow of the femur and tibia of female C57BL/6 mice (Jackson Labs; Ages 1–2 months) as described previously [22, 23]. HSCs were identified as the Lin^−^Sca-1^+^c-kit^+^ (LSK) fraction by incubating the cells with the following cocktail of antibodies (eBioscience San Diego, CA): PE-conjugated Sca-1 (1:100 dilution), APC-Cy7 conjugated c-kit (1:100 dilution), and a 1:100 dilution of a FITC-conjugated Lineage (Lin) cocktail (CD5, B220, Mac-1, CD8a, Gr-1, Ter-119). The LSK cell fraction was sorted using a BD FACS Aria II flow cytometer (BD FACS Diva software) and collected in PBS/FBS on ice for immediate use.

### Cell culture and surface antigen expression analysis

5000 freshly sorted LSKs were seeded into 96 well plate wells, along with 300 μL of StemSpan SFEM media (Stem Cell technologies, Vancouver, Canada) containing 100 ng/mL of Stem Cell Factor (SCF), (Peprotech, NJ). Cell culture was maintained in the incubator at 37°C and 5% CO_2_. Cell phenotype was determined at day 1, 4, 7 and 9, post culture via flow cytometry. The cells were first washed with PBS/FBS containing 0.1% sodium azide, centrifuged at 1400 rpm for 5 minutes, resuspended in 100 μL and stained with the following antibody cocktail (eBioscience): APC anti-CD34 (1:20 dilution, 60 mins), APC-Cy7 anti-c-kit (1:100, 30 mins), PE anti-Sca-1 (1:100, 30 mins), PE-Cy7 anti-CD16/32 (1:100, 30 mins), FITC-anti lineage cocktail (CD5, B220, Mac-1, CD8a, Gr-1, Ter-119, 1:100, 30 mins). Cells were identified as LSK (Lin^−^c-Kit^+^ Sca-1^+^), Common Myeloid Progenitors (CMP) (Lin^−^c-Kit^+^ Sca-1^–^ CD34^+^ CD16/32^–^) or Long Term HSC (LT-HSC) (LSK·CD34^–^·Flk2^–^), Short Term HSC (ST-HSC) (LSK·CD34^+^·Flk2^–^), and Multipotent Progenitor (MPP) (LSK·CD34^+^·Flk2^+^). All remaining cells were identified as ‘Terminal’ for the purposes of the model. All analysis was done on the BD LSRII flow cytometer (BD Bioscience) and the FCS Express software.

### ELISA to determine SCF concentration in culture

SCF concentration in culture was determined by performing ELISA (R&D systems, Minneapolis, MN) on the supernatant media extracted during FACS analysis. Analysis was performed at end points for the condition of no media exchange, as per manufacturer’s protocol. This provided the maximum possible SCF consumption rate for the purposes of model estimations (**Table A in S1 File**).

### Computational model using STELLA and Berkeley Madonna

The computational software STELLA (isee systems, NH) was used to generate a mathematical representation of the cell culture system using graphical means, while curve fitting of the model to experimental data was done using the software Berkeley Madonna.

#### 3-cell (LSK–CMP–Terminal) computational model

1. The 3 cell groupings (LSK, CMP, Terminal) were designated as individual stocks in culture, where each stock had inputs (proliferation and/or differentiation from previous state) and outputs (apoptosis and/or differentiation into next state). The general differential equation for each state can be given as:
  $$\frac{d{Cell}_{i}}{dt}={Cell}_{i\;proliferation}-{Cell}_{i+j\;differentiation}+{Cell}_{i-j\;differentiation}-{Cell}_{i\;apoptosis}$$
  Where *i+j* and *i-j* are the subsequent or prior cell states in the differentiation hierarchy.
2. Media was designated as a stock, with the input and output set as a pulse function with a frequency corresponding to the frequency of media exchange (2 or 10). Media volume was set to 300 μL and remained constant during the culture period.
3. SCF was designed as a stock with an input concentration of 100 ng/mL. The input was also set as a pulse function with a frequency corresponding to the frequency of media exchange (2 or 10). SCF output was a combination of a pulse function (where SCF was refreshed corresponding to media changes) and SCF consumed by the cells during culture. With a constant cellular consumption rate, SCF consumed by the cells (*K_SCF_consumption*) was:
  $$SCF_{consumed}=K_{SCF_{consumption}}*\#cells$$
  SCF Consumption rate was estimated based on the experimentally observed SCF values at various time points (**Table A in S1 File**). For simplicity, this rate was kept constant in the model, but within experimentally observed limits.
4. Nutrient availability within the media (labeled GC for Glucose) was defined as a function similar to SCF with an input concentration of 10 mM and a constant cellular consumption rate (*K_GC_consumption*) such that the amount consumed by the cells was:
  $${GC}_{consumed}=K_{{GC}_{consumption}}*\#cells$$
  Model values for this rate were based off previously observed consumption rates [24].
5. Four groups of cell-secreted biomolecules were classified as proliferation stimulators (*ProS*), proliferation inhibitors (*ProI*), differentiation stimulators (*DiffS*), differentiation inhibitors (*DiffI*). Initial models defined biomolecule secretion from each cell sub-set at a constant rate. Therefore, the amount of the respective biomolecules in culture is the sum of individual secretion factors, such that:
  $$Biomolecule\;(ng)=\sum_{i=1}^{n}c_{i}*\#cells$$
  Where *c*_*i*_ is the secretion rate (ng/cell/day) for each cell type and is a function of the active concentration of the secreted cytokines, thus forming a feedback loop. For simplification, the total number of LSK and CMP cells at any given time were summed and denoted ‘Progenitors’. Therefore, the amount of biomolecule secreted by the system is given by:
  $$Biomolecule\;(ng)=c_{i}*Progenitors+c_{j}*Terminal$$
  Where each cell secretion rate was given as:
  $$c_{i}=m_{i}e^{\left(-[biomolecule]\right)}$$
  Where *m*_*i*_ is the secretion rate (ng/cell/day). In the model, ratios of the stimulators vs. the inhibitors were defined as:
  $$R_{d}=\frac{DiffS}{DiffI}\;R_{p}=\frac{ProS}{ProI}$$
6. Cell apoptosis was a function of GC concentration in the media for each cell type such that the death rates (DR) was given by:
  $${Cell_{i}}_{DR}=\frac{{Cell_{i}}_{DRmax}}{1+d_{i}*[GC]}$$
  Here, *Cell*_*i_DRmax*_ is the maximum biological death rate for that cell type and *d*_*i*_ is a constant.
7. Progenitor populations have the ability to self-renew and the fraction of self-renewing progenitors at any given time (*f*_*i*_) is based off a modified Monod equation and is given by:
  $$f_{i}=\frac{f_{i\;max}}{1+R_{d}+s_{i}*[SCF]}$$
  Where *f*_*i max*_ is the maximum self-renewal capacity (≤ 1) and *s*_*i*_ are constants. Since the denominator is always ≥ 0, as both *R*_*d*_ and the SCF concentration ([*SCF*]) are positive, the self-renewing fraction of any cell type *f*_*i*_ is always ≤ *f*_*i max*_.
8. The proliferation rate of each cell type was given by *PR*_*i*_ and was defined by a Gaussian-type function [21]:
  $${PR}_{i}={PR}_{i\;max}*(1-e^{-([SCF]*R_{p})})$$
  Where *PR*_*i max*_ is the maximum proliferation rate.
9. A quintessential property of stem cells is their ability to be in a quiescent state, where they are not involved in any biological activity such as proliferation or differentiation. The LSK state was assigned a quiescent fraction *q*, which was determined by the degree of differentiation being induced in the cells in culture and given another Gaussian-type function:
  $$q=q_{max}*e^{-(R_{d})}$$
  Where *q*_*max*_ is the maximum quiescent fraction. Here, it is hypothesized that the quiescent fraction of the stem cells drops in response to a rise in the concentration of differentiation stimulators (and hence *R*_*d*_).
10. The differentiation rate for cell state *i* to cell state *j* is given by *DR*_*itoj*_ (#cells/cell/day) and is a constant.
11. Finally, we assign a fraction to LSK cells that transition directly into the Terminal state, while skipping CMPs as *j*. This dynamic is dictated by feedback provided by the Terminal cells via concentration of the secreted differentiation stimulators: c3*#Terminal cells. This active concentration is denoted *T*_*d*_, such that:
  $$j=j_{max}*e^{-({[T}_{d}])}$$
  Where *j*_*max*_ (≤ 1) and is a constant. Increasing numbers of Terminal cell population trigger a negative feedback response within the LSK differentiation dynamic that drops the differentiating fraction (*j*). Therefore, as [*T*_*d*_] increases, $e^{-[T_{d}]}$ decreases and the jump fraction over longer periods reduces.

#### 5-cell (LT-HSC–ST-HSC–MPP–CMP–Terminal) computational model

The computational model for the 5-state system was similar to the 3-state model with the following exceptions:

1. Proliferation rates for each cell type was a constant
2. Cell secretion rates were constant
3. Jump fractions were assigned to 2 different cell states: *j*_*ST-HSCtoTerminal*_ and *j*_*MPPtoTerminal*_
4. Quiescent fractions were assigned to 3 different cell states: *q*_*LT-HSC*_, *q*_*ST-HSC*_, *q*_*MPP*_

The full system of differential equations developed based on these criteria for both 3-state and 5-state models, along with parameter values are provided in the S1 file.

## Results

### Dynamic growth profiles of early and late progenitors indicate complex culture feedback

**Fig 1:**
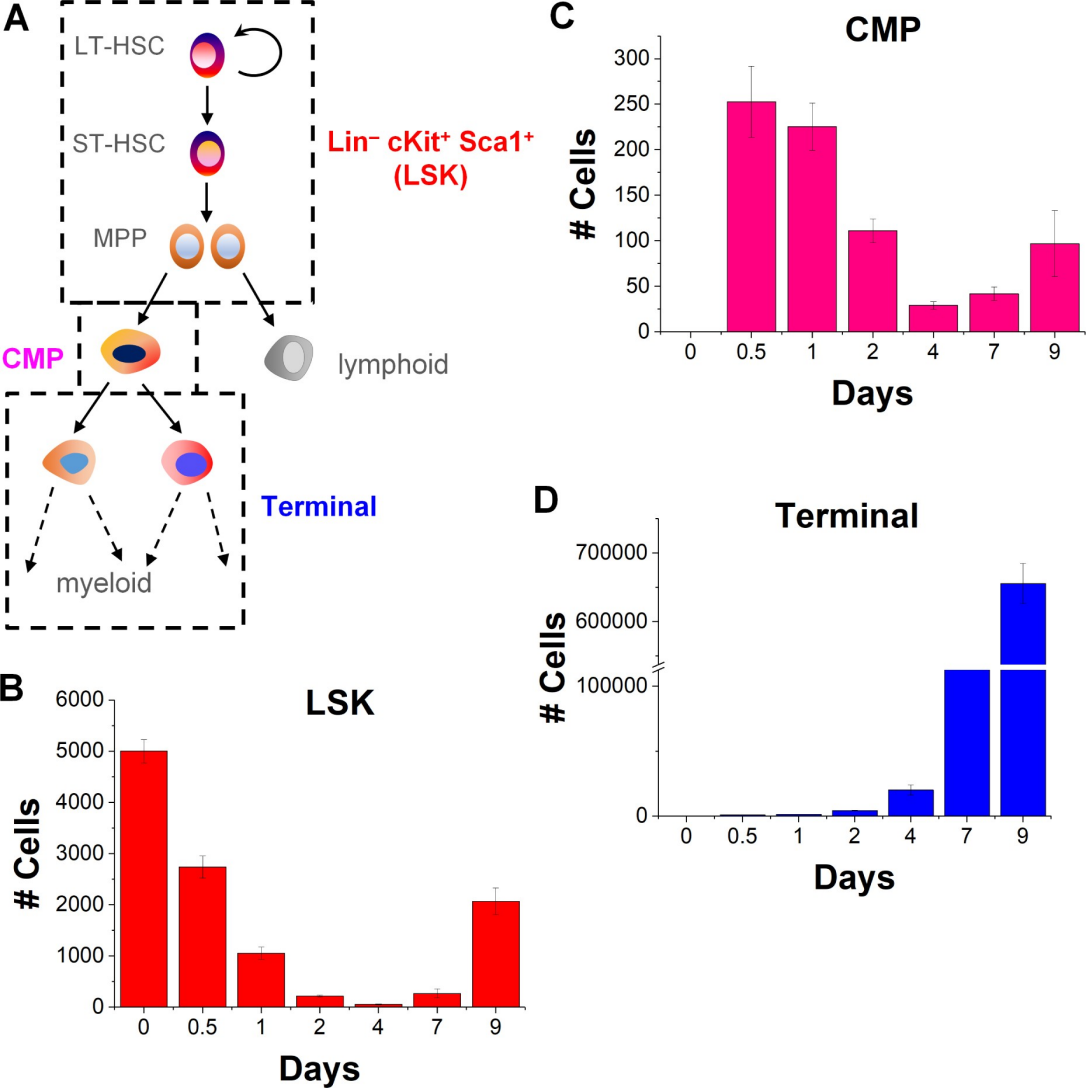
FACS analysis of cell progenitor populations over time. (A) Schematic of the classical HSC differentiation hierarchy where early progenitors (identified as LSK) differentiate into CMPs before maturing into fully developed myeloid cells (Terminal). (C-D) Total number of each cell sub-population (LSK, CMP, Terminal) in culture over time. While the LSK cells recover some of their numbers, CMP population show variable kinetics but overall low numbers, and Terminal cells grow at an exponential rate. Error bars represent standard error of mean.

The classical model of HSC hierarchy involves stepwise differentiation of early long-term repopulating progenitors into late oligopotent progenitors and eventually terminally differentiated mature myeloid or lymphoid cells [25]. Primary murine HSCs were identified on the basis of their surface antigens via flow cytometry (FACS) as early progenitors (LSK: Lineage^−^cKit^+^ Sca1^+^) cells, late progenitors (common myeloid progenitors (CMP: Lin^–^c-kit^+^Sca1^–^CD34^+^CD16/32^–^), and terminally differentiated (Terminal: remainder of hematopoietic cells) (**Fig 1A, S1A Fig**)[25] and analyzed over a 9-day culture period. With a starting population of only LSK cells, the LSK fractions showed a biphasic behavior with a steady decline through day 4 followed by a rapid increase through day 9. The CMP population exhibited a more dynamic profile with multiple rises and falls in their overall numbers over time. Finally, Terminal cells increased rapidly in number through the end of the experiment. Critically, the magnitude of changes associated with each cell population were vastly different in scale (**Fig 1C–1E**). While the Terminals cells reached a peak population of 6 x 10^5^, LSK cells oscillated in number between 10^2^ and 2 x 10^3^, and CMPs maintained a variable but overall low numbers (<300) throughout. The relative numbers and time-scales of these responses highlight that hematopoietic sub-populations may display vastly different kinetics despite being in the same culture.

### CMPs represent a transient state in the hematopoietic differentiation hierarchy

**Fig 2:**
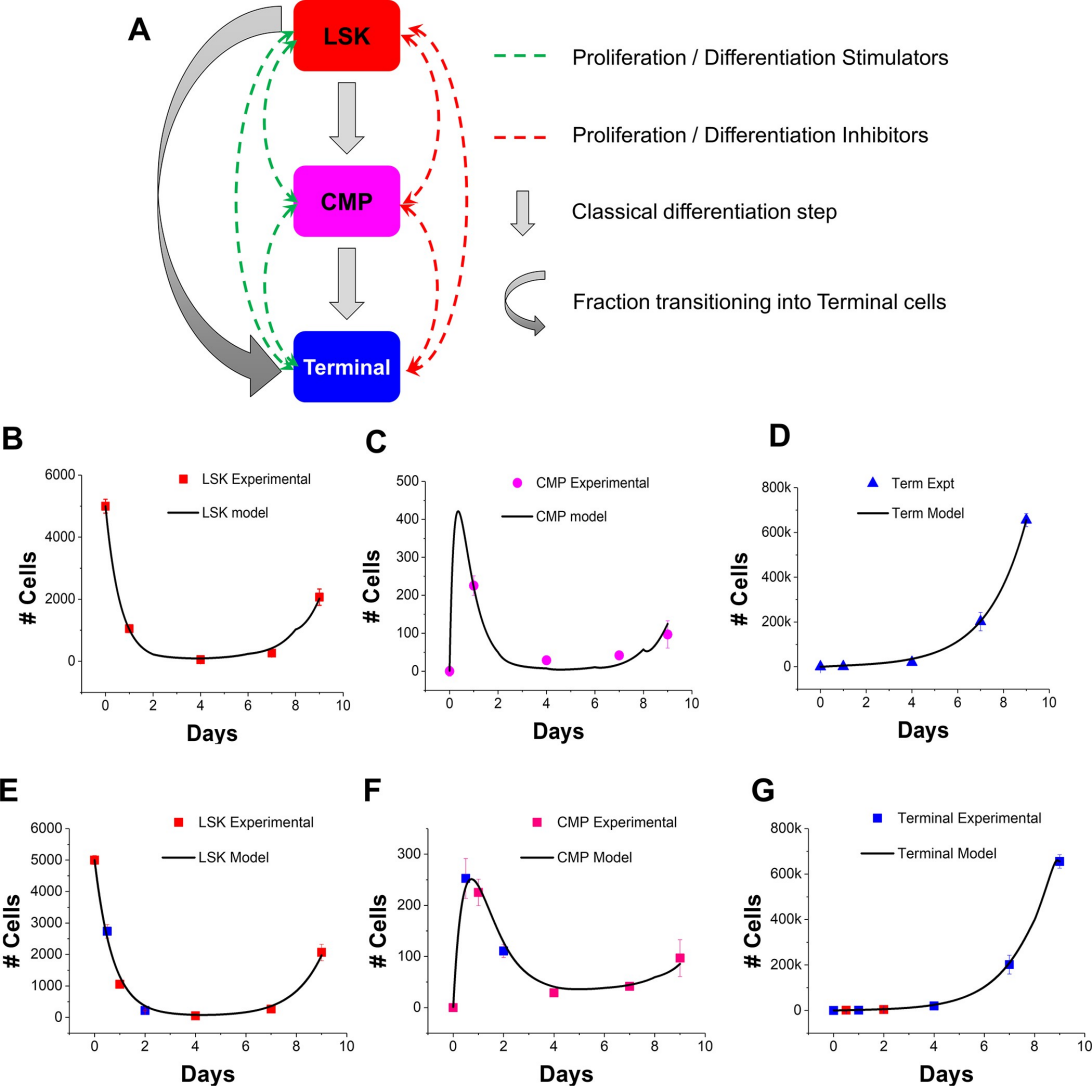
Computational set up of a 3-state cell model and experimental verification. (A) A 3-state computational model featuring the LSK, CMP and Terminal hematopoietic cells. Culture dynamics is influenced via feedback from cell-secreted biomolecules that stimulate and/or inhibit the proliferation and differentiation rates of each cell type. Additionally, a fraction of LSKs would be available to transition directly into Terminal (‘jump’). (B-D) The model simulation is able to capture the dynamics and scale of the experimental data for all 3 cell profiles (B: LSK; C: CMP; D: Terminal). The initial model further predicted a peak in CMP population prior to day 1 of culture. (E-G) Further experiments were performed to capture data before and after the 1 day data point (12hr; 48hr) for all three cell sub-populations (E: LSK; F: CMP; G: Terminal) to substantiate the initial model predictions. Error bars represent standard error of mean.

We developed a first order differential equation model describing the kinetics of growth and early myeloid specification for a 3-cell state model of early hematopoietic differentiation **(Fig 2A)**. Cell-secreted biomolecules that directly or indirectly influence cell kinetics such as proliferation, apoptosis, and differentiation were categorized into 4 types: differentiation stimulators (*DiffS*) and inhibitors (*DiffI*), and proliferation stimulators (*ProS*) and inhibitors (*ProI*). Cell secretion rates are designed to dynamically respond to active biomolecule concentrations through a feedback mechanism such that the culture system, and the model, naturally adjusts to temporal changes expected due to cell secretions and cell-cell interactions. We expressed the temporal changes in kinetic parameters such as the self-renewal fractions (*f*_*LSK*_, *f*_*CMP*_), quiescent fraction of the early progenitors (*q*_*LSK*_), and cell proliferation rates (*PR*_*LSK*_, *PR*_*CMP*_, *PR*_*Terminal*_) as functions of biomolecule concentrations. Given recent evidence [26] for a two-tier model of HSC hierarchy during blood cell development rather than the classical step-wise differentiation process, we also introduce the concept of a state ‘jump’. Here a fraction of LSK cells (*j*_*LSK*_) circumvent the intermediate CMP state and differentiate directly into Terminal cells, making the CMP state more transient in the developmental process of mature myeloid phenotypes.

Model simulations were able to successfully capture changes in cell numbers for all 3-cell sub-populations, providing a strong validation for the underlying kinetics described (**Fig 2B–2D**). Interestingly, the model predicted the existence of an early spike in the CMP population prior to day 1 of culture (the first time point at which we gathered experimental data). Given the high correlation between model fit and experimental data, we considered the possibility that the peak was not a model anomaly but existed at a hitherto untested data point. We verified the existence of this peak by repeating experiments to gather data points both before (12h) and after (2 days) the original day 1 data point (**Fig 2E–2G**). Excitingly, the model successfully predicted the peak increase for the CMP sub-population prior to the day 1 experimental data point, while accurately representing the kinetics of LSK and Terminal cells throughout the 12h to 2-day culture period. Notably, the presence of a jump fraction (*j*_*LSK*_) was crucial in obtaining a high degree of correlation between model predictions and experimental data. Eliminating this fraction and its corresponding kinetics led to a poor experimental-model fit **(S2 Fig)**. This suggests a more involved differentiation process than the traditionally accepted stepwise differentiation pathway for mature cell development. While even a relatively simple HSC liquid culture displays complex cell growth profiles, the mathematical model was able to robustly capture key features of its dynamics and nuances.

### Model response to culture perturbations and analysis of parameter values

**Fig 3:**
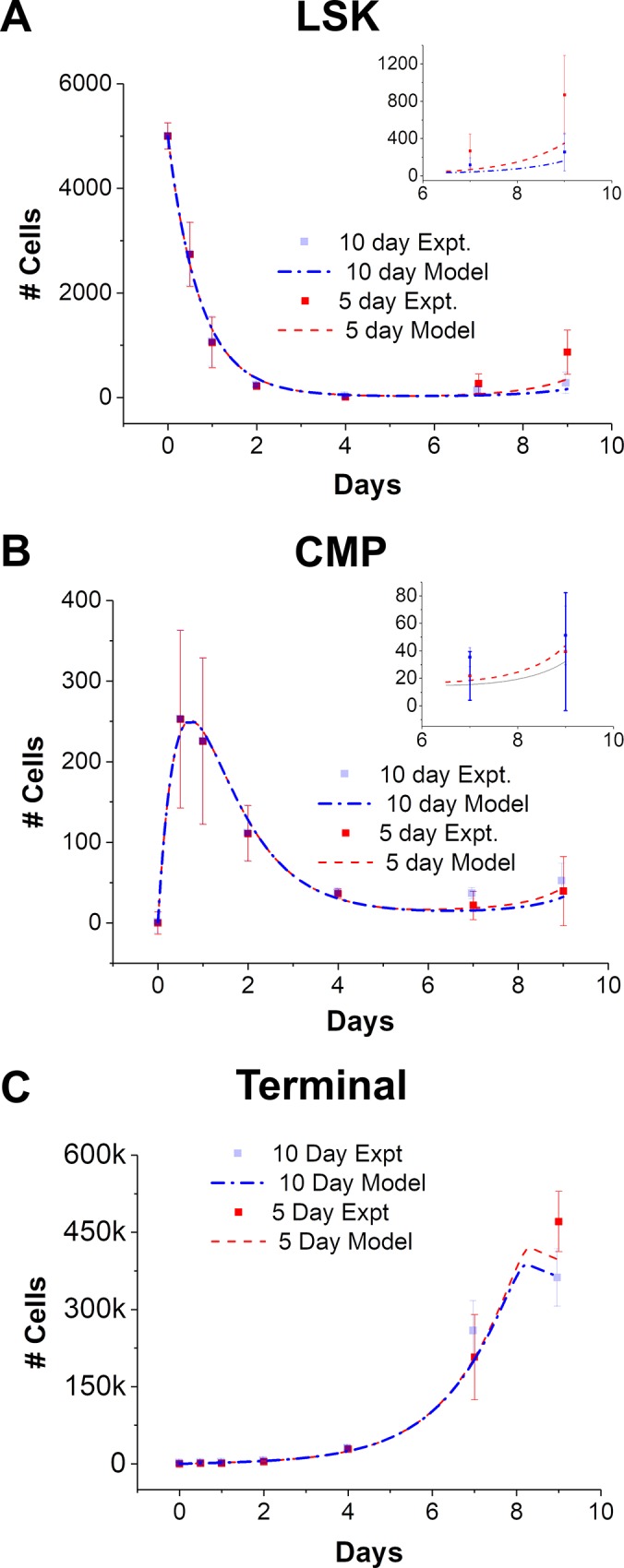
Model fit and predictions for intermediate media exchange frequencies. (A—C) Undisturbed culture conditions over the 9 day period (denoted as 10 day), led to a drop in all cell populations as compared to the 2-day media exchange. Model parameters were optimized to obtain a high degree of correlation with experimental data simply by changing media frequency change in the model to 10 days. The model was able to predict the system response to a 5-day media exchange for all the 3 cell populations, without any parameter optimization. Subtle differences in cell numbers were captured over long term culture. Inset: a close up of the model and data fit between days 7–9. Error bars represent standard deviation.

We leveraged model capabilities to predict the system’s response to changes in culture conditions. Given that signaling feedback has been previously shown to be vital in regulating HSC fate,[18, 27, 28] we explored the consequence of altering the timeframe for media exchange changes and its effect on the balance between self-renewal vs. differentiation. Under conditions of no media exchange (10 day media change), experiments indicate that the recovery of both LSK and CMP populations after the initial decline (at day 4) is reduced compared to media exchange every 2 days. Not surprisingly, expansion of Terminal cells is drastically reduced with no media exchange. The model could successfully reflect these experimental profiles simply by changing the media frequency parameter, further underlining the efficacy of the model. In order to further validate the capacity of the model to adapt to multiple culture conditions, we generated model predictions for an intermediate (5-day) media exchange frequency. These predictions suggested that while the temporal profile of the cell populations remains unchanged, slight variations in absolute cell numbers were to be expected, particularly at longer times **(Fig 3A–3C, inset)**. Experimental results matched model predictions of increased number of LSK, CMP, and Terminal cells relative to no media exchange.

We established temporal profiles of kinetic parameters involved in cell proliferation and differentiation. Here, the model suggests the self-renewing fractions of LSK and CMP increase with longer culture periods (**S3A Fig**), presumably with decreased differentiation and increased proliferative rates. Not surprisingly, the number of quiescent LSKs remain relatively low while a steady fraction of LSKs continue transitioning into Terminal cells over time. The importance of cell-secreted biomolecules in regulating feedback-mediated cell kinetics is observed through their respective profiles **(S3B and S3C Fig)**. Higher concentrations of predicted differentiation stimulators during early culture periods promote Terminal cell expansion, while the increase in differentiation inhibitors secreted by the mature progeny contribute to the observed increase in progenitor numbers. Interestingly, the inflection point for this change is around day 4 and corresponds to the changes in cell profiles. The proliferation rates for each cell type remain relatively constant through most of the 9-day culture period **(S3D Fig),** while the increase in apoptosis rates at longer time points correlate with a decrease in SCF and nutrient availability owing to higher cell numbers (**S3E and S3F Fig**).

### Cell differentiation is driven by the proliferative short-term HSCs, while long-term HSCs remain quiescent in culture

**Fig 4:**
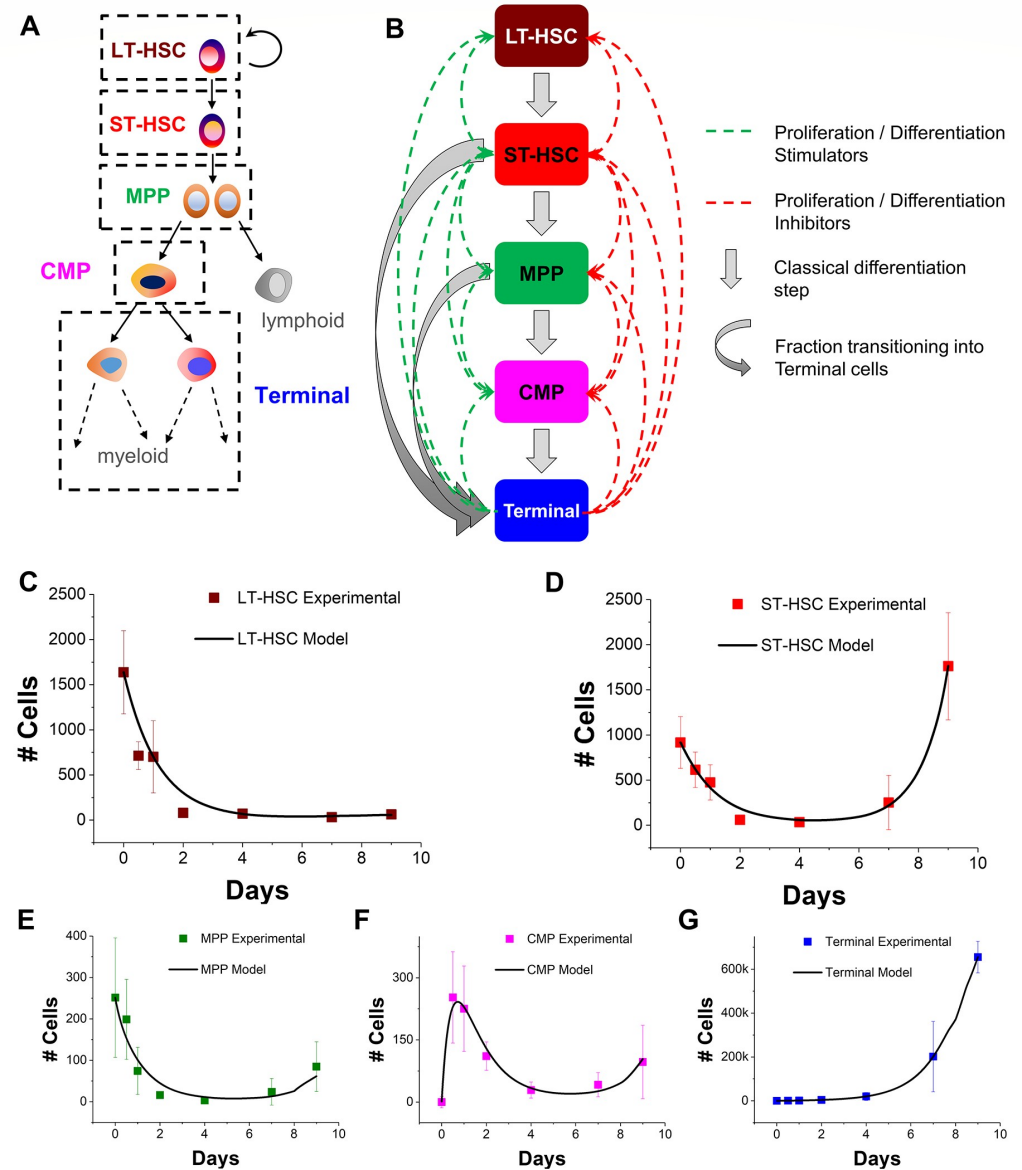
A five state model framework for HSC lineage specification. (A) A schematic of the classical HSC differentiation hierarchy where long-term progenitors (LT-HSC) differentiate into short-term (ST-HSC) progenitors, then multipotent (MPP), then common myeloid progenitors (CMP) before maturing into fully developed myeloid cells (Terminal). (B) A 5-state computational model featuring the LT-HSC, ST-HSC, MPP, CMP, and Terminal cell types. Culture dynamics may be influenced via feedback from cell-secreted biomolecules that stimulate and/or inhibit the proliferation and differentiation rates of each cell type. Additionally, a fraction of ST-HSCs and MPPs would be available to transition directly into Terminal cells without going through CMP differentiation (‘jump’). (C-G) The model simulation is able to capture the dynamics and scale of the experimental data for all 5 cell profiles (C: LT-HSC; D: ST-HSC; E: MPP; F: CMP; G: Terminal) over a 9 day period. Error bars represent standard deviation.

Building upon the 3-state system, we increased the model complexity by separating the heterogeneous LSK population into 3 discrete stages: the most primitive long-term repopulating HSC (LT-HSC), the short-term repopulating HSC (ST-HSC), and the multipotent progenitors (MPP) **(Fig 4A)** [28, 29]. Our model for this 5-cell state system (LT-HSC, ST, HSC, MPP, CMP, Terminal) relied on a series of linked 1^st^ order differential equations that expanded on the 3-state system described previously. Jump fractions were included between ST-HSC (*j*_*ST-HSCtoTerm*_) and MPP (*j*_*MPPtoTerm*_) cells transitioning directly into Terminal cells **(Fig 4B)**. Based on kinetic profiles of the 3-cell model, proliferation, apoptosis, and cell secretion rates were set constant for all cell types. We observe that the LT-HSC population reduces significantly over the culture period while the MPP and CMP fractions have similar growth kinetics. The bulk of the early progenitor activity was compartmentalized in the ST-HSC sub-populations, where these cells showed biphasic behavior similar to that observed for the larger LSK fraction in the 3-state model (**S4 Fig**). The 5-state model was able to successfully fit the growth curves for all the five cell types simultaneously (**Fig 4C–4G**). The model predicts that similar to the 3-state model the jump fractions remain relatively constant over the 9-day culture period and with roughly equal contributions from ST-HSCs and MPPs (**S5B Fig**). The model also predicts a high fraction of LT-HSCs remain quiescent while ST-HSCs show elevated self-renewing fraction in culture (**S5A and S5C Fig**). Similar to the 3-state model, the presence of jump fractions was critical for a high degree of model-data fit. Model simulations failed to capture the growth kinetics of all cell populations, over-estimating the number of early progenitors due to the absente jump fractions, while underestimating the Terminal cell populations possibly owing to delayed differentiation (**S6 Fig**).

### Identification of high-impact parameters

System stability in response to perturbations in individual parameter values is an essential part of the model and presents an opportunity to identify critical parameters to exploit in future experimental cultures. From an engineering perspective focused on developing a more guided HSC culture platform, we endeavored to identify biological or kinetic parameters that have the greatest impact on each cell state. Local parameter sensitivity analysis was performed by defining the sensitivity S where [21]:

$$S=\frac{\langle \Delta O/O\rangle }{\langle \Delta P/P\rangle }$$

**Fig 5:**
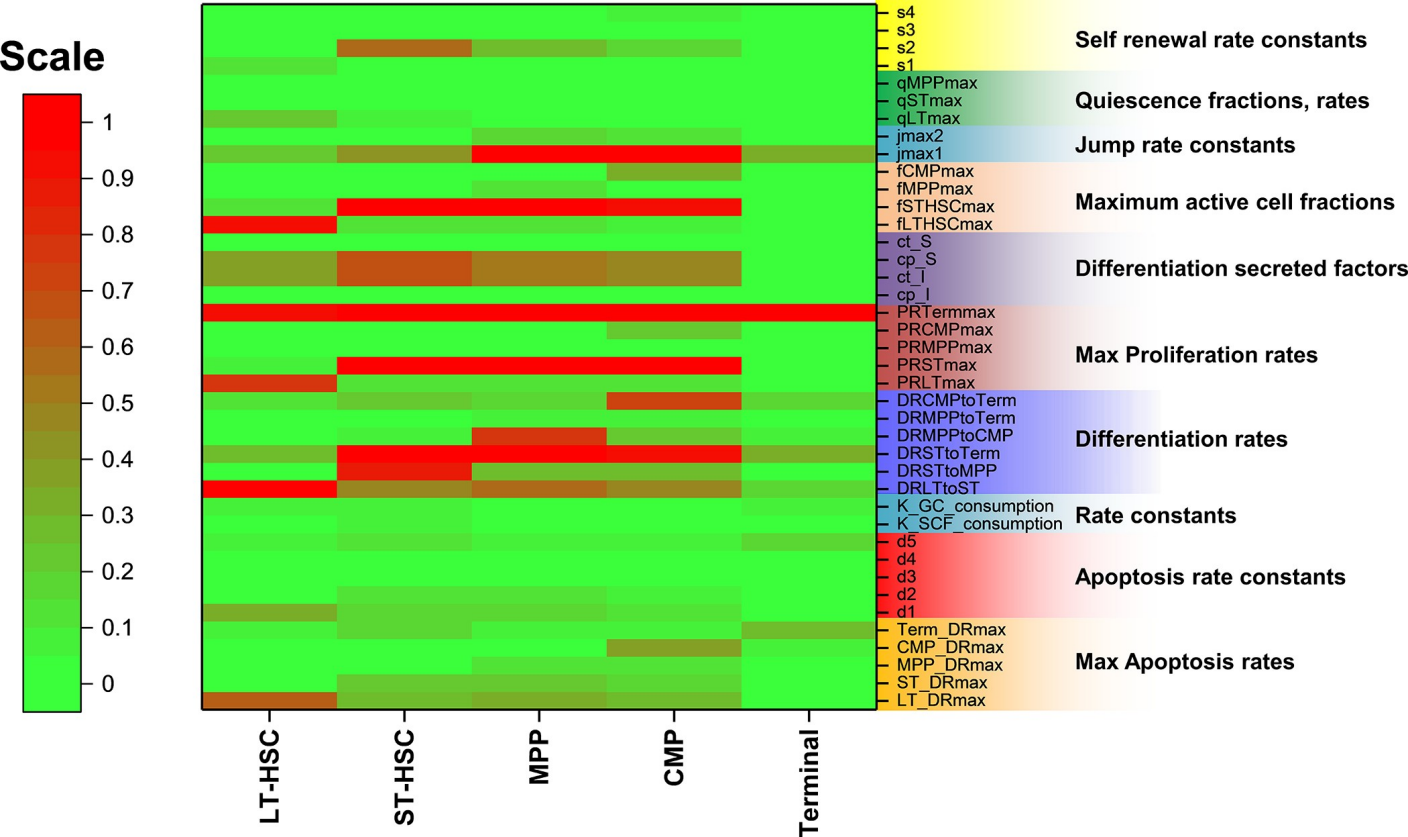
Parameter sensitivity matrix of the 5-state HSC differentiation model, broken down by cell state (LT-HSC, ST-HSC, MPP, CMP, Terminal) and type of model parameter. Red nodes in the matrix indicate that model sensitivity is >1% for a 1% change in parameter values. This is indicative of high parameter sensitivity and system instability, but also design parameters for future experimental optimization. Terminal cells, on account of their large and heterogeneous populations, are relatively insensitive to several model parameters except their proliferation rates (PRTermmax).

Where ΔO is the change in output value, O is the original output value, ΔP is the change in parameter value (1%) and P is the original parameter value. The resulting sensitivity S is thus the average of individual sensitivities at each time point. This analysis produces an *i* (cell states) x *j* (parameters) matrix for S values, which were then projected onto a heat map, developed for both the 3-state (**S7 Fig**) and 5-state model (**Fig 5**). Overall, the Terminal cell populations were the least sensitive to parameter perturbation for both model structures, possibly on account of their large numbers and cellular heterogeneity that was not accounted for in the model. The most number of high-impact parameters were observed for ST-HSCs, MPPs, and CMPs, that possessed both a dynamic growth profile and were the early drivers of cell growth in culture. Kinetic parameters such as maximum proliferation rates (*PR*_*ST_max*_, PR_Terminal_max_), differentiation rates, self-renewing fractions (*f*_*ST-HSC-max*_), and select secretion constants had a large impact on system response. Interestingly, the impact of these parameters was consistently observed across all cell populations. The 3-cell model elicited a similar parameter sensitivity response with consistencies observed for both LSK and CMP populations.

To address our long-term goal of developing engineering system to selectively expand more primitive HSC populations in vitro, we closely examined cell-specific sensitivities in order to identify the temporal trends in parameter sensitivities. The sensitivity profile varied widely across all conditions, with sensitivities either increasing, decreasing, or changing dynamically over time. Increasing parameter sensitivities are an indication that model stability is weakened over longer culture periods. Representative profiles of parameter sensitivities (**S8 Fig**) highlight the highly complex system response underway, as can be seen from the non-linear profiles across all parameters and cell populations. Interestingly, inflection points for all cell types are consistently observed around days 3–5, where the corresponding cell growth kinetics are the most volatile. These data suggest that accurate experimental determination of high-impact parameters is vital to developing an accurate and stable model. Sensitivity information of this nature can be potentially utilized to dynamically alter targeted parameters (based on high sensitivity) at specific time points (based on their temporal response) to modulate cell fate.

## Discussion

The ability to identify key parameters that dominate culture kinetics and utilizing them to engineer optimal conditions for stem cell expansion is important for future advances in hematopoietic stem cell biomanufacturing. Bone marrow transplants that rely on high numbers of repopulating hematopoietic stem and progenitor cells for marrow reconstitution could benefit from such strategic cell expansion. Our efforts build on key findings in the literature. Using a mathematical model, *Kirouac et al* demonstrated umbilical cord blood (UCB) Lin^−^cells progenitor cell fate is directly integrated with cell-cell signaling mechanisms and cell-secreted biomolecules [20, 21]. *Csaszar et al*. employed an integrated computational and experimental approach to increase the yield of HSCs within a closed culture system by regulating the negative paracrine signaling feedback via a controlled media dilution method [19]. Building upon previous modeling frameworks we developed a multi-state (3 and 5) differentiation system to capture essential elements of the feedback dynamics that govern HSC progenitor vs. mature cell growth in vitro. Dynamic changes in these cell populations suggest that much of this process is dictated by the endogenous cell-secreted biomolecules broadly categorized to influence cell differentiation and proliferation. While the kinetics of subsets of these biomolecule groups is likely much more nuanced and complex, our classification was sufficient to adequately capture the observed population profiles *in vitro*. The role of inhibitory factors secreted by differentiating hematopoietic cell populations on progenitor cell differentiation and proliferation arrest has been previously observed [13]. *Marciniak-Czochra et al*. proposed parallel multi-compartment models for HSC differentiation to examine the relationship between stem cell self-renewal and proliferation rate and the expansion of progenitor populations. Their results elegantly identify the role of early progenitor population self-renewal in steady state hematopoiesis [30]. Notably, our model can successfully replicate these observations, highlighting the complex relationship between signaling feedback and cell fate determination.

The concept of a non-hierarchical, discrete development of lymphoid and myeloid progenitors through HSC differentiation has been gaining traction. The existence of the proposed adult lymphoid-primed multipotent progenitors (LMPP) that lack erythroid and megakaryocyte lineage specification question the hierarchical relevance of common myeloid (CMP) and lymphoid (CLP) progenitors in the classical HSC differentiation model[31, 32]. *Notta et al*. therefore proposed a two-tier hierarchy of hematopoietic development in adult hematopoiesis[26] where the main stem cell compartment remains multipotent while the progenitors are instead unipotent and strongly committed to the myeloid or lymphoid lineage. These findings support the need to revise the classical step-wise transitions from early to late progenitors both experimentally and in the model. Excitingly, our results show unequivocally that the cell state ‘jump’ from LSK to Terminal (in the 3-cell model) or from ST-HSC and MPP to Terminal (in the 5-cell model) is a critical parameter required to capture the kinetics of the experimental data with a computational model. Experimentally, CMP populations remained relatively small throughout the 9-day culture, making a step-wise transition from LSK to CMP and ultimately to Terminal cells not sufficient to accurately describe observed growth rates. The dependence of this transient (jump) fraction on cellular feedback is also evident in the case where media is not replenished over the 9 day culture period, leading to the accumulation of differentiation and proliferation inhibitors within the system (**S10 Fig**). It is important to note that all cell populations are detected via surface antigen expression profiles at discrete time points. Consequently, nuances of single cell differentiation process may diverge due to transience of gene or surface antigen expression patterns. It is likely that understanding more deeply population dynamics of cohorts of hematopoietic cells, classified via a series of constants in this model, may involve more complex culture kinetics. Therefore, a more deterministic partial differentiation equation model (PDE) may be required for future efforts to gain more comprehensive understanding of the entire HSC differentiation hierarchy. Such models have been employed to investigate the role of cytokine signaling on cancer cell activity[33], cell cycle response to therapeutics [34], and the human-microbiome symbiosis[35]. Interestingly, stochastic models have also been employed to study a wider range of biological systems and multi-cell populations [36–38]. So while the absolute kinetics dictating hematopoietic cell behavior is expected to be far more complex, our study provides insight regarding tradeoff in early hematopoietic cell differentiation as well as conforms to the recently observed non-hierarchical HSC model [26], suggesting it provides an important first validation of such modeling approaches.

Sensitivity analysis provides key insight into model stability and identification of parameters that have the biggest influence on culture kinetics. They represent master descriptors of optimal culture conditions with stem cell biomanufacturing potential. The parameter sensitivities observed are an indication of model stability; while low sensitivity values indicate minimal response to system perturbations the retained parameters remain crucial to model output. Our model is sensitive to many parameters at varying degrees, the most prominent being the proliferation and differentiation rates of individual cell populations (**Fig 5, S7 Fig**). We assessed parameter impact roughly split into an early and late-stage response in culture. A rapid initial response implies that a higher degree of control during the early culture phase is important for regulating the final outcome. Experimental determination of some of the unknown parameters could potentially improve model stability and provide a more reliable model construct. While the differentiation rates in the model are assumed constant over the 9-day culture period, the dynamic cellular feedback observed hints to a more non-linear and interdependent rate kinetics. A more stable model allows us to better establish such kinetics and enable a model-parameter system capable of accurately predicting cell behavior for a wider range of culture conditions. Although the mathematical expressions and the number of parameters used in our model can be subject to modification, the approach described here highlights the possibility of describing complicated cell culture dynamics with a high degree of accuracy. A reduction in the number of variables would reduce model complexity, but at the cost of kinetic information such as temporal shifts in proliferation or apoptosis rates, dynamically changing self-renewal vs. quiescent fractions, and signaling cascades between cell populations. The dependence of cell response on biomolecular feedback identified by this model presents an opportunity to tailor culture systems response in a manner similar to that exploited to create bioreactor paradigms for HSC culture [27], and by our lab to alter the diffusive transport of paracrine vs. autocrine signals within a 3D hydrogel to influence HSC fate specification [39]. The work described here provides a facile tool to inform efforts profiling shifts in the secreted biomolecules as a means to identify factors that can be added exogenously or perturbed endogenously to boost early hematopoietic cell expansion.

In order to be clinically applicable, large-scale expansion of hematopoietic populations requires biomanufacturing capabilities with a high degree of control over early progenitor populations (HSCs, MPPs). Such tools will likely require the addition of supporting niche cells, exogenous biomolecules, and the presence of a biomaterial matrix [40, 41] within a dynamic culture environment. Experimentally determining the individual and combinatorial effects of all external factors will likely be an extremely expensive and time intensive process. Modeling efforts, such as the approached described here, can provide a powerful tool to facilitate rapid assessment and optimization of culture parameters to direct HSC proliferation and differentiation. Here, our findings that HSC hierarchy and lineage differentiation *in vitro* is not wholly stepwise but rather follows grouped developmental routes is an important attribute to be considered for future experimental and computational studies.

## Conclusions

We employ an integrated computational and experimental approach to successfully describe *in vitro* culture kinetics of murine hematopoietic stem cells. Used as a prognostic tool, the model was able to capture the multi-scale population shifts in multiple hematopoietic subpopulations and predict cell response to different culture conditions in a means that could be used to tailor experimental conditions for selective expansion of discrete hematopoietic sub-populations. We demonstrate that the CMP population represents a transient state within the HSC hierarchy and much of the downstream differentiation is driven by rapid differentiation of primarily short term repopulating HSC progenitor populations. These observations are consistent with the recently proposed tiered rather than step-wise HSC differentiation model. Finally, via sensitivity analyses we identify culture parameters, such as proliferation and biomolecule secretion rates, most strongly associated with the observed experimental responses as a means to generate more complex HSC culture models optimized for targeted cell expansion.

## Supporting information

S1 File Supporting information and tables. (DOCX)

S1 Fig Cell sorting strategy for hematopoietic cell populations. (A) Singlets selected from the population are first gated into live (DAPI^–^) cells, followed by gating into Lin^−^(FITC) cells. The LSK population is gated across c-kit^+^ (PE) and Sca1^–^ (APC-Cy7) and a Pre-CMP selection is made for the c-kit^+^ and Sca1^–^_,_ followed by the CMP gate Lin^–^ckit^+^Sca1^–^. The LSK population is further gated into LT-HSC (Flk2^–^ CD34^–^), ST-HSC (Flk2^–^ CD34^+^) and MPP (Flk2^+^ CD34^+^). (B) Representative FACS plots of the early progenitor populations across 9 days in culture, with the fractions denoting their relative populations from the LSK sub fraction. Overall, the ST-HSC population increases most substantially whereas the size of LT-HSC and MPP populations remain small throughout.
(TIF)

S2 Fig Model predictions in the absence of the jump fraction. When the *j*_*LSK*_ value is set to 0, the model is unable to capture the experimental data for (A) LSK, (B) CMP, and (C) Terminal cells, particularly at long time periods. The lack of early jump fraction leads to a predicted over-accumulation of the CMP cells and reduced size of the Terminal cell population. Error bars represent standard error of mean.
(TIFF)

S3 Fig Model-predicted profiles of multiple culture parameters that influence the expansion of each cell sub-set. (A) The self-renewing fractions of LSK (*f*_*LSK*_) and CMP (*f*_*CMP*_) cells, the fraction of LSKs that ‘jump’ to Terminal cells while bypassing the CMP stage (*j*) and the relatively low quiescent numbers of the early progenitors (*q*) in culture. A relatively high fraction of LSK cells transition into Terminal cells even during day 9 of culture. (B, C) The concentrations of proliferation and differentiation stimulators and inhibitors in the culture media dictate the shift of cell fractions from differentiation to proliferation and is a function of cell-secreted cytokines. (D) Proliferation rates of each cell type remain largely constant over the culture period. (E) death (or apoptosis) rates of all cell types are predicted to remain constant while there is a high level of SCF and nutrient (Glucose availability) and increases with nutrient depletion (F) SCF and Glucoase (GC) content is replenished every 2 days due to media exchange and, decreasing rapidly with increasing cell numbers.
(TIFF)

S4 Fig Total number of each cell type in culture over time. (A) The LT-HSC population declines over the entire culture period. (B) ST-HSCs recovers after day 4 of culture in a manner similar to the overall LSK cell growth observed in the 3-cell state model. (C) Similar kinetics are observed for MPPs and CMPs. (D) An exponential increase in the Terminal cell population is seen. Error bars represent standard error of mean.
(TIF)

S5 Fig Predicted culture kinetics for the 5-state cell model. (A) self-renewing fractions of each sub-set rise steadily over time, also evidenced by the rising cell numbers. (B) fraction transitioning directly from ST-HSC or MPP to Terminal cells (jump). A higher number of ST-HSCs ‘jump’ to Terminal compared to MPPs, however each fraction is maintained over the entire culture period. (C) A higher fraction of LT-HSCs are predicted to remain quiescent while the more mature ST-HSC and MPP cells display a reduced quiescent capability. (D) The concentrations of differentiation stimulators and inhibitors in the culture media change dynamically over time, and is a function of cell-secreted cytokines. (E) Similar to the 3-state model, SCF and Glucose (GC) content is replenished every 2 days due to media exchange and, decreasing rapidly with increasing cell numbers. (F) Death (or apoptosis) rates of all cell types are predicted to remain constant while there is a high level of SCF and nutrient (Glucose availability) and increases with nutrient depletion.
(TIFF)

S6 Fig Model predictions in the absence of the jump fraction. When the *j*_*STHSCtoTerm*_ and *j*_*MPPtoTerm*_ values are set to 0, the model is unable to capture the experimental profiles for all cell populations. (A–E). The ST-HSC, MPP, and CMP populations exceed experimental observations, while Terminal cells are underpopulated due to lower initial differentiating cell numbers.
(TIFF)

S7 Fig Parameter sensitivity matrix of the 3-state HSC differentiation model, broken down by cell state (LSK, CMP, Terminal) and type of model parameter. Red nodes in the matrix indicate that model sensitivity is >1% for a 1% change in parameter values. This is indicative of high parameter sensitivity and system instability, but also design parameters for future experimental optimization. Similar to the 5-state model, Terminal cells, on account of their large and heterogeneous populations are relatively insensitive to several model parameters except their proliferation rates (PRTermmax).
(TIFF)

S8 Fig Representative temporal profiles of parameter sensitivities. (A-C) For the 3 state model, changes in sensitivity for select parameters for each cell type indicate that the system response is highly non-linear. While the impact of some parameters steadily rises over time (Apoptosis_LSK_, Prolif_CMP_ etc.), others plateau or decrease over time. (D-F). These dynamic profiles are also observed for the 5-state model. Cell state response to these factors can help us identify the positive or negative impact of specific parameters over time, and whether culture modulation can further help regulate system response.
(TIFF)

S9 Fig Graphical representation of the model in STELLA. (A) Schematic of the overall differentiation process with input and output flows associated with each cell type. The inputs correspond to increase in cell population (proliferation, differentiation from previous state) whereas outputs correspond to decrease in cell population (apoptosis, differentiation into next state). Rates associated with each flow are described in the equations given in the Supplemental section. (B) Schematic of the 3 exogenous soluble components of the system: Media, SCF, nutrient availability (denoted as GC for Glucose). Exchanging the media replenishes both components and is controlled by the parameter ‘change frequency’. (C) Concentrations of groups of biomolecules (DiffS, DiffI, ProS, and ProI) are governed by the number of cells and a constant secretion rate associated with each cell type (c1 –c12) which dictate the self-renewing fractions (DiffS, DiffI) and the proliferation rates (ProS, ProI) for all cell types.
(TIF)

S10 Fig Concentration of proliferation and differentiation inhibitors for the condition where media exchange does not take place over the 10 day period. (TIFF)
