# Supplementary material for: A computational model of feedback-mediated hematopoietic stem cell differentiation in vitro
Source: PLoS One. 2019 Mar 1;14(3):e0212502. doi: 10.1371/journal.pone.0212502 (PMC6396932; doi:10.1371/journal.pone.0212502)
Supplement: S1 File — (DOCX) [file pone.0212502.s001.docx]

# Supporting Information and tables

# Materials and Methods

## 3-cell differential equation model

The full system of differential equations for the 3-cell (LSK – CMP – Terminal) model is as follows:

$$\frac{dLSK}{dt}=LSK_{in}-LSK_{to}CMP-LSK_{to}Term-LSK_{dead}$$

$$\frac{dCMP}{dt}=CMP_{in}+LSK_{to}CMP-LSK_{to}Term-{CMP}_{dead}$$

$$\frac{dTerminal}{dt}={Terminal}_{in}+{CMP}_{to}Terminal+{LSK}_{to}Terminal-{Terminal}_{dead}$$

$$\frac{dSCF}{dt}={SCF}_{in}-SCF_{out}-SCF_{consumed}$$

$$\frac{dGC}{dt}=GC_{in}-GC_{out}-GC_{consumed}$$

$$\frac{dDiffI}{dt}={DiffI}_{in}-{DiffI}_{out}$$

$$\frac{dDiffS}{dt}={DiffS}_{in}-{DiffS}_{out}$$

$$\frac{dProI}{dt}={ProI}_{in}-{ProI}_{out}$$

$$\frac{dProS}{dt}={ProS}_{in}-{ProS}_{out}$$

Proliferation rates:

$$LSK_{in}=LSK*PR_{LSK}*f_{1}$$

$${CMP}_{in}=CMP*PR_{CMP}*f_{2}$$

$${Terminal}_{in}=Terminal*PR_{Terminal}$$

$$PR_{LSK}= {PR}_{LSKmax}*(1-e^{-\left( \left[ SCF \right]*R_{p} \right)})$$

$$PR_{CMP}= {PR}_{CMPmax}*(1-e^{-\left( \left[ SCF \right]*R_{p} \right)})$$

$$PR_{Terminal}= {PR}_{Terminalmax}*(1-e^{-\left( \left[ SCF \right]*R_{p} \right)})$$

Self-renewing, quiescent, and jump fractions:

$$f_{1}= \frac{f_{1 max}}{1+R_{d}+s_{1}*[SCF]}$$

$$f_{2}= \frac{f_{2 max}}{1+R_{d}+s_{2}*[SCF]}$$

$$q= q_{max}*e^{-(R_{d})}$$

$$j=j_{max}*e^{-({[T}_{d}])}$$

Differentiation rates:

$$LSK_{to}CMP=LSK*\left( 1-f_{1}-q-j \right)*DR_{LSKtoCMP}$$

$${CMP}_{to}Terminal=CMP*\left( 1-f_{2} \right)*DR_{CMPtoTerminal}$$

$$LSK_{to}Terminal=LSK*j*DR_{LSKtoTerminal}$$

Apoptosis rates:

$$LSK_{dead}=LSK*LSK_{DR}$$

$${CMP}_{dead}=CMP*{CMP}_{DR}$$

$${Terminal}_{dead}=Terminal*{Terminal}_{DR}$$

$$LSK_{DR}=\frac{LSK_{DRmax}}{1+d_{1}*[GC]}$$

$$CMP_{DR}=\frac{CMP_{DRmax}}{1+d_{2}*[GC]}$$

$$Terminal_{DR}=\frac{Terminal_{DRmax}}{1+d_{3}*[GC]}$$

SCF and GC rates:

$$SCF_{in}=Desired SCF Concentration*Pulse\left( 0.3,change\_frequency \right)$$

$$SCF_{out}=Pulse(SCF,change\_ frequency)$$

$$SCF_{consumed}=K_{SCF_{consumption}}*Total cells$$

$${GC}_{in}=Desired SCF Concentration*Pulse\left( 0.3,change\_frequency \right)$$

$${GC}_{out}=Pulse(SCF,change\_ frequency)$$

$${GC}_{consumed}=K_{{GC}_{consumption}}*Total cells$$

Cell-secreted biomolecule rates:

$$DiffS_{in}= c_{1}*Progenitor+ c_{5}*Terminal$$

$$DiffI_{in}= c_{2}*Progenitor+ c_{6}*Terminal$$

$$ProS_{in}= c_{3}*Progenitor+ c_{7}*Terminal$$

$$ProI_{in}= c_{4}*Progenitor+ c_{8}*Terminal$$

$${DiffS}_{out}=Pulse(DiffS,change\_ frequency)$$

$${DiffI}_{out}=Pulse(DiffI,change\_ frequency)$$

$${ProS}_{out}=Pulse(ProS,change\_ frequency)$$

$${ProI}_{out}=Pulse(ProI,change\_ frequency)$$

Where

$$c_{1}=m_{1}*e^{[DiffS]}$$

$$c_{2}=m_{2}*e^{[DiffI]}$$

$$c_{3}=m_{3}*e^{[ProS]}$$

$$c_{4}=m_{4}*e^{[ProI]}$$

$$c_{5}=m_{5}*e^{[DiffS]}$$

$$c_{6}=m_{6}*e^{[DiffI]}$$

$$c_{7}=m_{7}*e^{[ProS]}$$

$$c_{8}=m_{8}*e^{[ProI]}$$

$$Progenitor=LSK+CMP$$

Curve fitting of the model to experimental data for parameter estimation was done using the software Berkeley Madonna. The Runge-Kutta 4 integration method was used to estimate parameter values for a model-experimental data match giving the smallest root mean square error. The parameter values for the 3-state model (rounded to 3 decimal digits) are shown in **S2 Table**.

## 5-cell computational model using STELLA and Berkeley Madonna

The full system of differential equations for the 5-cell (LT-HSC – ST-HSC – MPP – CMP – Terminal) model is as follows:

$$\frac{dLTHSC}{dt}={LTHSC}_{in}-{LTHSC}_{to}STHSC-LSK_{dead}$$

$$\frac{dSTHSC}{dt}={STHSC}_{in}+LTHSC_{to}STHSC - {STHSC}_{to}MPP-STHSC_{to}Terminal-{STHSC}_{dead}$$

$$\frac{dMPP}{dt}={MPP}_{in}+{STHSC}_{to}MPP-{MPP}_{to}CMP-{MPP}_{to}Terminal-{MPP}_{dead}$$

$$\frac{dCMP}{dt}={CMP}_{in}+MPP_{to}CMP-{CMP}_{to}Terminal-{CMP}_{dead}$$

$$\frac{dTerminal}{dt}={Terminal}_{in}+{STHSC}_{to}Terminal+{MPP}_{to}Terminal+CMP_{to}Terminal-{Terminal}_{dead}$$

$$\frac{dSCF}{dt}={SCF}_{in}-SCF_{out}-SCF_{consumed}$$

$$\frac{dGC}{dt}=GC_{in}-GC_{out}-GC_{consumed}$$

$$\frac{dDiffI}{dt}={DiffI}_{in}-{DiffI}_{out}$$

$$\frac{dDiffS}{dt}={DiffS}_{in}-{DiffS}_{out}$$

Proliferation rates:

$${LTHSC}_{in}=LTHSC*PR_{LTHSC}*f_{LTHSC}$$

$${STHSC}_{in}=STHSC*PR_{STHSC}*f_{STHSC}$$

$${MPP}_{in}=MPP*PR_{MPP}*f_{MPP}$$

$${CMP}_{in}=CMP*PR_{CMP}*f_{CMP}$$

$${Terminal}_{in}=Terminal*PR_{Terminal}$$

Self-renewing, quiescent, and jump fractions:

$$f_{LTHSC}= \frac{f_{LTHSC max}}{1+R_{d}+s_{1}*[SCF]}$$

$$f_{STHSC}= \frac{f_{STHSC max}}{1+R_{d}+s_{2}*[SCF]}$$

$$f_{MPP}= \frac{f_{MPP max}}{1+R_{d}+s_{3}*[SCF]}$$

$$f_{CMP}= \frac{f_{CMP max}}{1+R_{d}+s_{4}*[SCF]}$$

$$q_{LTHSC}= q_{LTHSC max}*e^{-(R_{d})}$$

$$q_{STHSC}= q_{STHSC max}*e^{-(R_{d})}$$

$$q_{MPP}= q_{MPP max}*e^{-(R_{d})}$$

$$j_{STHSCtoTerminal}=j_{max1}*e^{-({[T}_{d}])}$$

$$j_{MPPtoTerminal}=j_{max2}*e^{-({[T}_{d}])}$$

Differentiation rates:

$${LTHSC}_{to}STHSC=LTHSC*\left( 1-f_{LTHSC}-q_{LTHSC} \right)*DR_{LTHSCtoSTHSC}$$

$${STHSC}_{to}MPP=STHSC*\left( 1-f_{STHSC}-j_{STHSCtoTerminal}-q_{STHSC} \right)*DR_{STHSCtoMPP}$$

$${STHSC}_{to}Terminal=STHSC*j_{STHSCtoTerminal}*DR_{STHSCtoMPP}$$

$${MPP}_{to}CMP=MPP*\left( 1-f_{MPP}-j_{MPPtoTerminal}-q_{MPP} \right)*DR_{MPPtoCMP}$$

$${MPP}_{to}Terminal=MPP*j_{MPPtoTerminal}*DR_{MPPtoTerminal}$$

$${CMP}_{to}Terminal=CMP*\left( 1-f_{CMP} \right)*DR_{CMPtoTerminal}$$

Apoptosis rates:

$${LTHSC}_{dead}=LTHSC*{LTHSC}_{DR}$$

$${STHSC}_{dead}=STHSC*{STHSC}_{DR}$$

$${MPP}_{dead}=MPP*{MPP}_{DR}$$

$${CMP}_{dead}=CMP*{CMP}_{DR}$$

$${Terminal}_{dead}=Terminal*{Terminal}_{DR}$$

$${LTHSC}_{DR}=\frac{{LTHSC}_{DRmax}}{1+d_{1}*[GC]}$$

$${STHSC}_{DR}=\frac{{STHSC}_{DRmax}}{1+d_{2}*[GC]}$$

$${MPP}_{DR}=\frac{{MPP}_{DRmax}}{1+d_{3}*[GC]}$$

$${CMP}_{DR}=\frac{CMP_{DRmax}}{1+d_{4}*[GC]}$$

$$Terminal_{DR}=\frac{Terminal_{DRmax}}{1+d_{5}*[GC]}$$

SCF and GC rates:

$$SCF_{in}=Desired SCF Concentration*Pulse\left( 0.3,change\_frequency \right)$$

$$SCF_{out}=Pulse(SCF,change\_ frequency)$$

$$SCF_{consumed}=K_{SCF_{consumption}}*Total cells$$

$${GC}_{in}=Desired SCF Concentration*Pulse\left( 0.3,change\_frequency \right)$$

$${GC}_{out}=Pulse(SCF,change\_ frequency)$$

$${GC}_{consumed}=K_{{GC}_{consumption}}*Total cells$$

Cell-secreted biomolecule rates:

$$DiffS_{in}= c_{pS}*Progenitor+ c_{tS}*Terminal$$

$$DiffI_{in}= c_{pI}*Progenitor+ c_{tI}*Terminal$$

$${DiffS}_{out}=Pulse(DiffS,change\_ frequency)$$

$${DiffI}_{out}=Pulse(DiffI,change\_ frequency)$$

$$Progenitor=LTHSC+STHSC+MPP+ CMP$$

Where *c_pS_*, *c_tS_*, *c_pI_*, and *c_tI_* are constants. Similar to the 3-state model, curve fitting and parameter estimation was performed using Berkeley Madonna and the Runge-Kutta 4 integration method. The parameter values for the 5-state model (rounded to 3 decimal digits) are shown in **S3 Table**.

## Tables

| **Day** | **SCF concentration**  **(ng/mL)** | **SD** |
| --- | --- | --- |
| 2 | 104.8 | ± 4.41 |
| 4 | 99.9 | ± 0.58 |
| 5 | 94.04 | ± 6.13 |
| 7 | 75.75 | ± 11.23 |
| 9 | 41.1 | ± 5.95 |

**S1 Table. Concentration of SCF in supernatant media as determined via ELISA.** SCF consumption rate for the purposes of the model was determined based on the available SCF concentration and the number of cells in culture.

| **Parameter** | **Value** | **Units** |  | **Parameter** | **Value** | **Units** |
| --- | --- | --- | --- | --- | --- | --- |
| Desired_SCF_Conc | 100 | ng/mL |  | m6 | 7.04E-09 | ng/cell/day |
| Desired_Vol | 0.3 | mL |  | m7 | 4.48E-09 | ng/cell/day |
| Desired_GC_Conc | 10 | mM |  | m8 | 4.41E-10 | ng/cell/day |
| K_GC_consumption | 4.47E-12 | mol/cell/day |  | LSK_DR_max | 0.74 | 1/cell/day |
| K_SCF_consumption | 2.93E-05 | ng/cell/day |  | CMP_DR_max | 0.11 | 1/cell/day |
| PRLSKmax | 2.82 | 1/cell/day |  | Term_DR_max | 0.90 | 1/cell/day |
| PRCMPmax | 4.63 | 1/cell/day |  | d1 | 1.00 | - |
| PRTermmax | 0.82 | 1/cell/day |  | d2 | 43.75 | - |
| DRLSKtoCMP | 0.26 | 1/cell/day |  | d3 | 855.53 | - |
| DRCMPtoTerm | 1.36 | 1/cell/day |  | f1max | 0.82 | - |
| DRLSKtoTerm | 1.67 | 1/cell/day |  | f2max | 0.44 | - |
| m1 | 1.30E-07 | ng/cell/day |  | s1 | 1.00E-04 | - |
| m2 | 3.69E-10 | ng/cell/day |  | s2 | 0.0055 | - |
| m3 | 3.94E-09 | ng/cell/day |  | jmax | 0.27 | - |
| m4 | 2.06E-09 | ng/cell/day |  | qmax | 0.050 | - |
| m5 | 6.32E-10 | ng/cell/day |  |  |  |  |

**S2 Table. Parameter values and their associated rate units for the 3-state (LSK – CMP – Terminal) model of cell culture.** Parameter values differ depending on the frequency of media exchange and are estimated by fitting the corresponding data sets to the model.

| **Parameter** | **Value** | **Units** |  | **Parameter** | **Value** | **Units** |
| --- | --- | --- | --- | --- | --- | --- |
| Change_frequency | 2 | Days |  | DRMPPtoTerm | 1.54 | 1/cell/day |
| Desired_SCF_Conc | 100 | ng/mL |  | DRSTtoTerm | 3.102 | 1/cell/day |
| Desired_Vol | 0.3 | mL |  | fCMPmax | 0.344 | - |
| Desired_GC_Conc | 10 | mM |  | fLTHSCmax | 0.271 | - |
| LT_DRmax | 0.357 | 1/cell/day |  | fMPPmax | 0.312 | - |
| ST_DRmax | 0.333 | 1/cell/day |  | fSTHSCmax | 0.786 | - |
| MPP_DRmax | 0.64 | 1/cell/day |  | jmax1 | 0.307 | - |
| CMP_DRmax | 0.589 | 1/cell/day |  | jmax2 | 0.212 | - |
| Term_DRmax | 0.389 | 1/cell/day |  | s1 | 0.0024 | - |
| d1 | 137.87 | - |  | s2 | 0.00529 | - |
| d2 | 219.14 | - |  | s3 | 0.0071 | - |
| d3 | 36.08 | - |  | s4 | 0.00325 | - |
| d4 | 1.027 | - |  | K_SCF_consumption | 2.09E-05 | ng/cell/day |
| d5 | 541.56 | - |  | K_GC_consumption | 7.95E-12 | mol/cell/day |
| cp_I | 4.26E-10 | ng/cell/day |  | PRCMPmax | 2.881 | 1/cell/day |
| ct_I | 6.59E-09 | ng/cell/day |  | PRLTmax | 3.22 | 1/cell/day |
| cp_S | 2.93E-07 | ng/cell/day |  | PRMPPmax | 1.56 | 1/cell/day |
| ct_S | 1.00E-11 | ng/cell/day |  | PRSTmax | 4.639 | 1/cell/day |
| DRCMPtoTerm | 1.512 | 1/cell/day |  | PRTermmax | 0.836 | 1/cell/day |
| DRLTtoST | 0.702 | 1/cell/day |  | qLTmax | 0.469 | - |
| DRMPPtoCMP | 5.03 | 1/cell/day |  | qMPPmax | 0.030 | - |
| DRSTtoMPP | 1.39 | 1/cell/day |  | qSTmax | 0.023 | - |

**S3 Table. Parameter values and their associated rate units for the 5-state (LT-HSC – ST-HSC – MPP – CMP – Terminal) model of cell culture.**
