# Supplementary figures and images for: A computational model of feedback-mediated hematopoietic stem cell differentiation in vitro
Source: PLoS One. 2019 Mar 1;14(3):e0212502. doi: 10.1371/journal.pone.0212502 (PMC6396932; doi:10.1371/journal.pone.0212502)

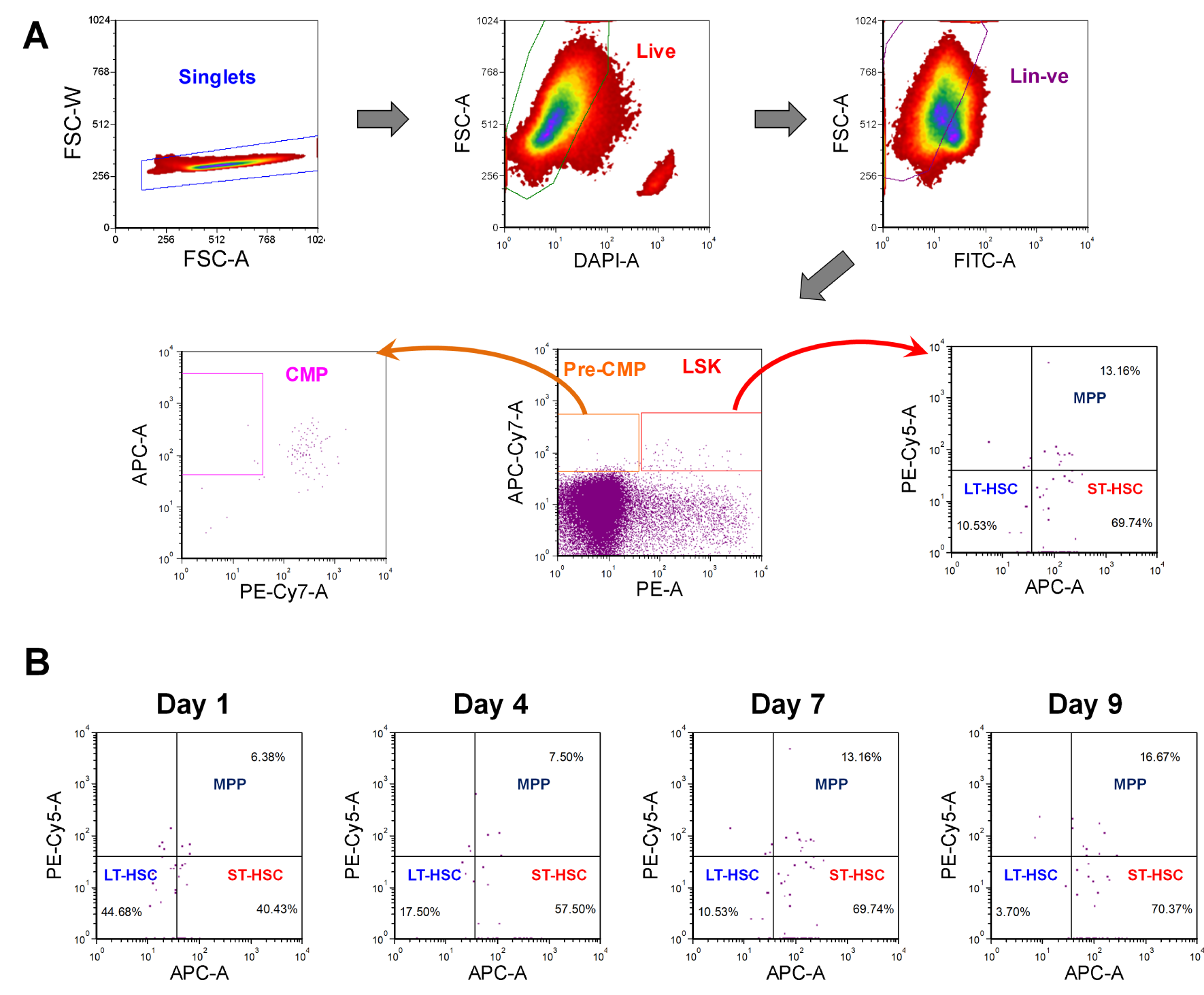

Supplement: S1 Fig — (A) Singlets selected from the population are first gated into live (DAPI–) cells, followed by gating into Lin−(FITC) cells. The LSK population is gated across c-kit+ (PE) and Sca1– (APC-Cy7) and a Pre-CMP selection is made for the c-kit+ and Sca1–, followed by the CMP gate Lin–ckit+Sca1–. The LSK population is further gated into LT-HSC (Flk2– CD34–), ST-HSC (Flk2– CD34+) and MPP (Flk2+ CD34+). (B) Representative FACS plots of the early progenitor populations across 9 days in culture, with the fractions denoting their relative populations from the LSK sub fraction. Overall, the ST-HSC population increases most substantially whereas the size of LT-HSC and MPP populations remain small throughout. (TIF) [file pone.0212502.s002.tif]

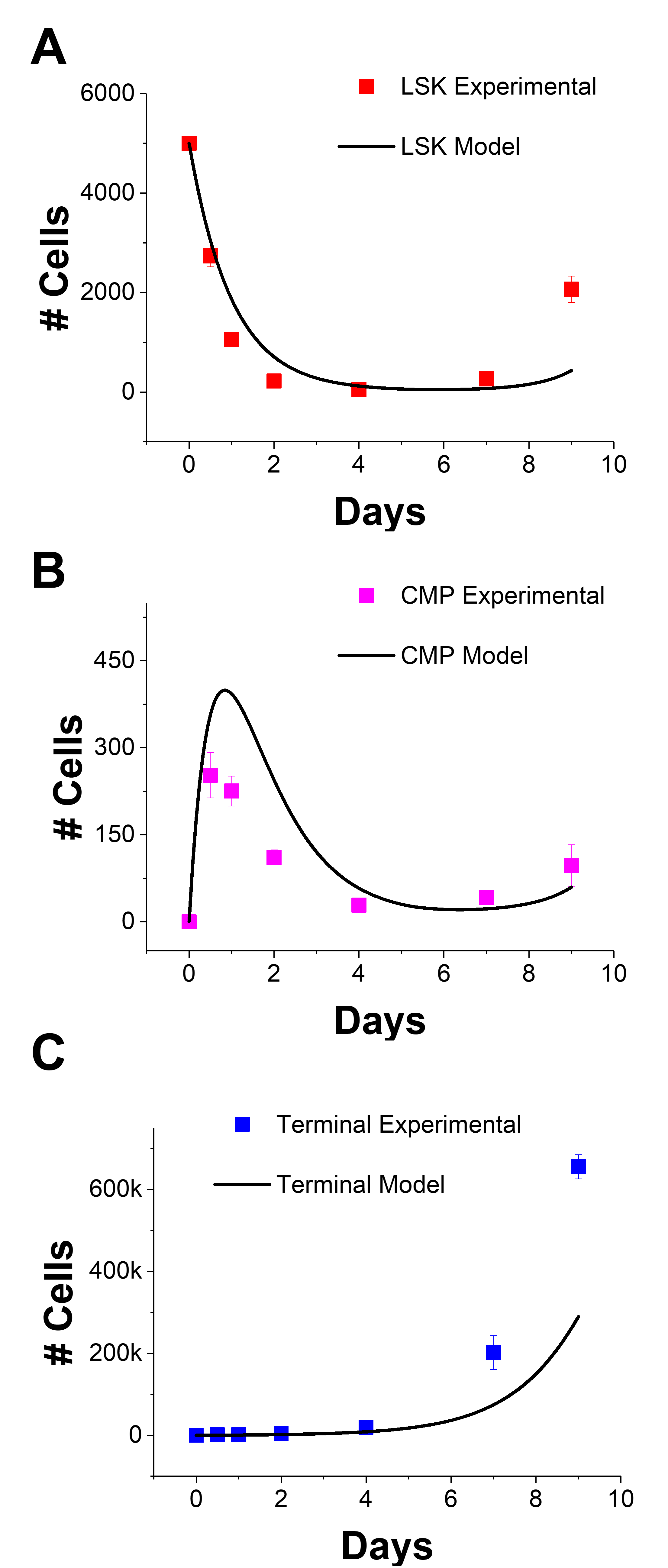

Supplement: S2 Fig — When the jLSK value is set to 0, the model is unable to capture the experimental data for (A) LSK, (B) CMP, and (C) Terminal cells, particularly at long time periods. The lack of early jump fraction leads to a predicted over-accumulation of the CMP cells and reduced size of the Terminal cell population. Error bars represent standard error of mean. (TIFF) [file pone.0212502.s003.tiff]

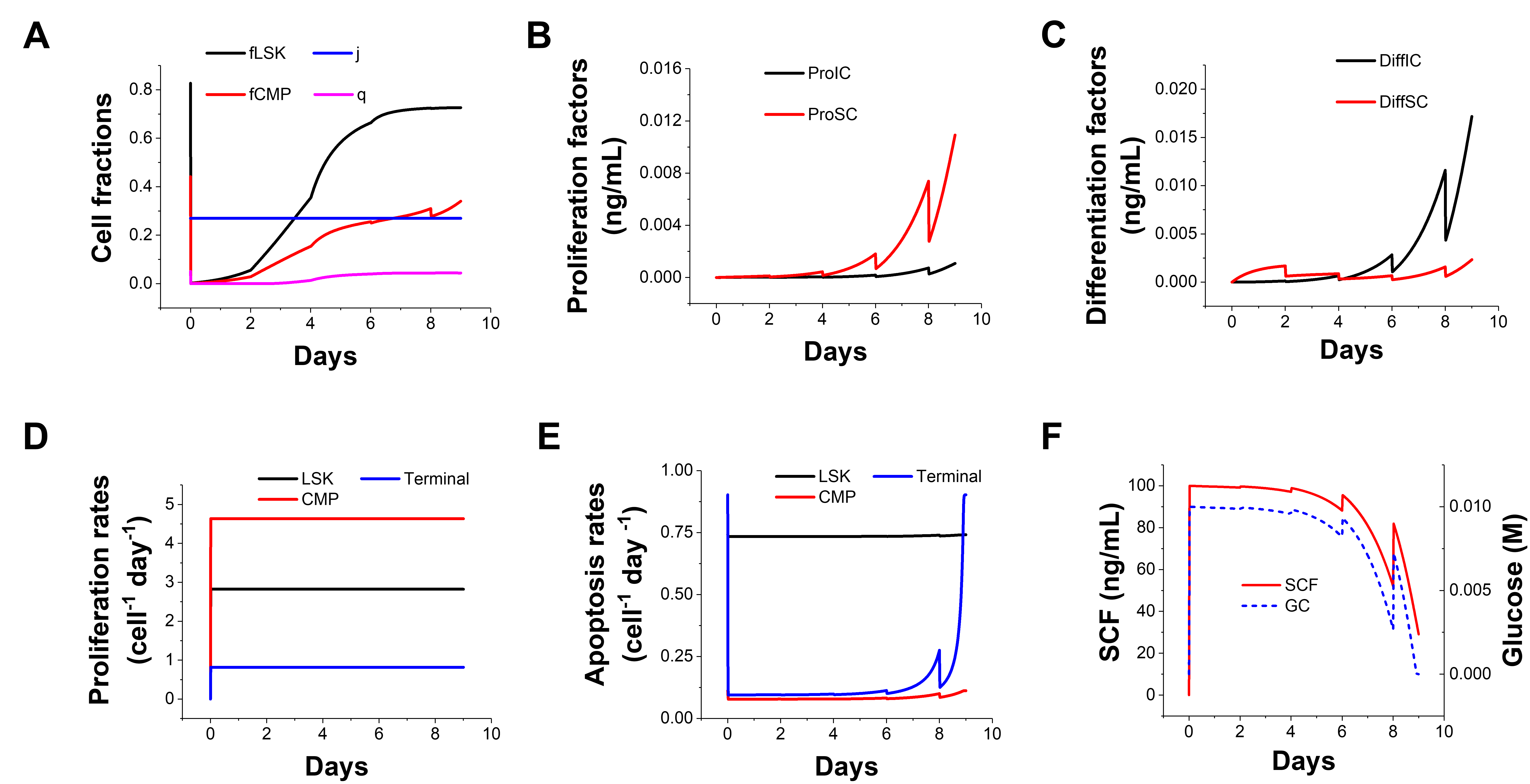

Supplement: S3 Fig — (A) The self-renewing fractions of LSK (fLSK) and CMP (fCMP) cells, the fraction of LSKs that ‘jump’ to Terminal cells while bypassing the CMP stage (j) and the relatively low quiescent numbers of the early progenitors (q) in culture. A relatively high fraction of LSK cells transition into Terminal cells even during day 9 of culture. (B, C) The concentrations of proliferation and differentiation stimulators and inhibitors in the culture media dictate the shift of cell fractions from differentiation to proliferation and is a function of cell-secreted cytokines. (D) Proliferation rates of each cell type remain largely constant over the culture period. (E) death (or apoptosis) rates of all cell types are predicted to remain constant while there is a high level of SCF and nutrient (Glucose availability) and increases with nutrient depletion (F) SCF and Glucoase (GC) content is replenished every 2 days due to media exchange and, decreasing rapidly with increasing cell numbers. (TIFF) [file pone.0212502.s004.tiff]

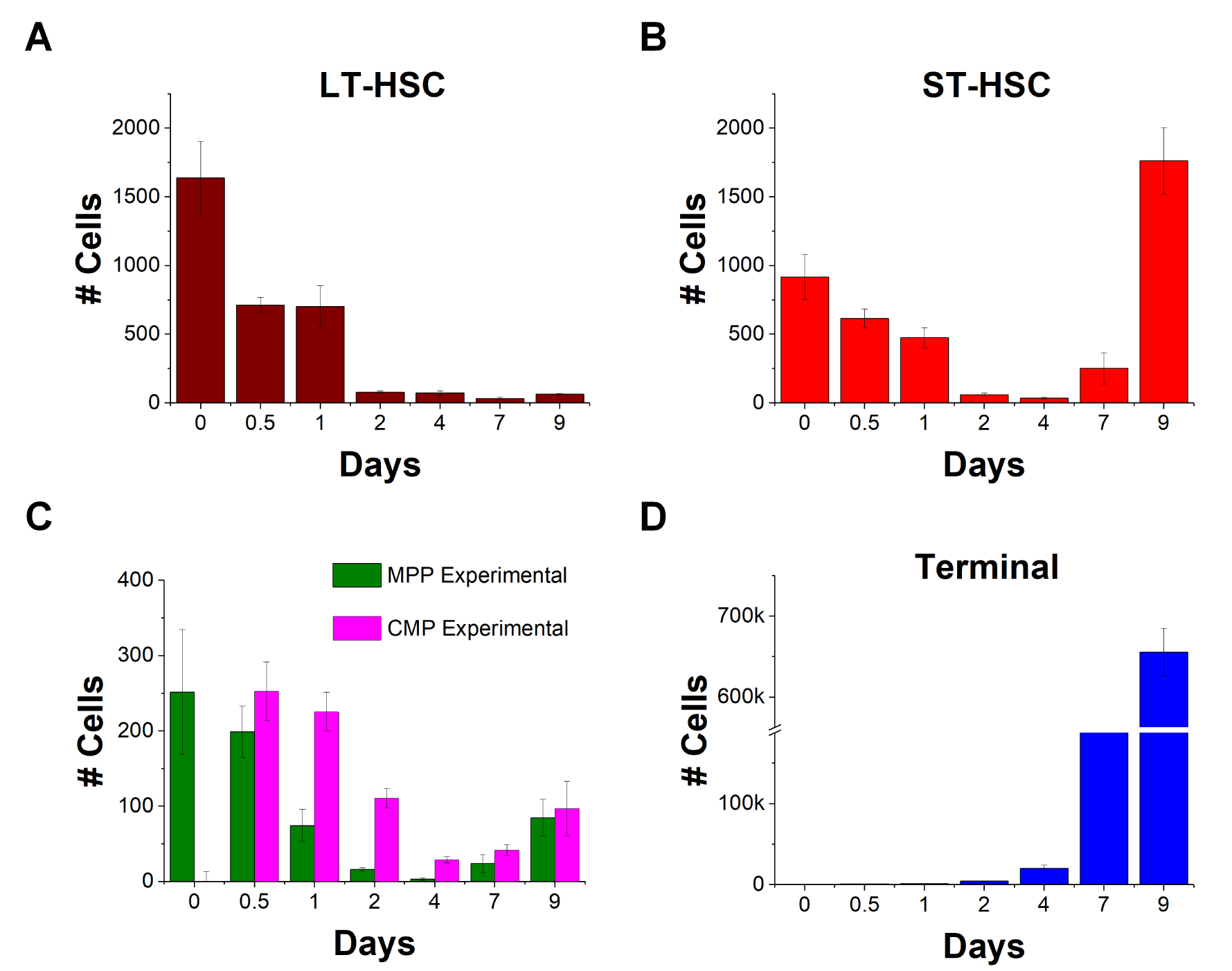

Supplement: S4 Fig — (A) The LT-HSC population declines over the entire culture period. (B) ST-HSCs recovers after day 4 of culture in a manner similar to the overall LSK cell growth observed in the 3-cell state model. (C) Similar kinetics are observed for MPPs and CMPs. (D) An exponential increase in the Terminal cell population is seen. Error bars represent standard error of mean. (TIF) [file pone.0212502.s005.tif]

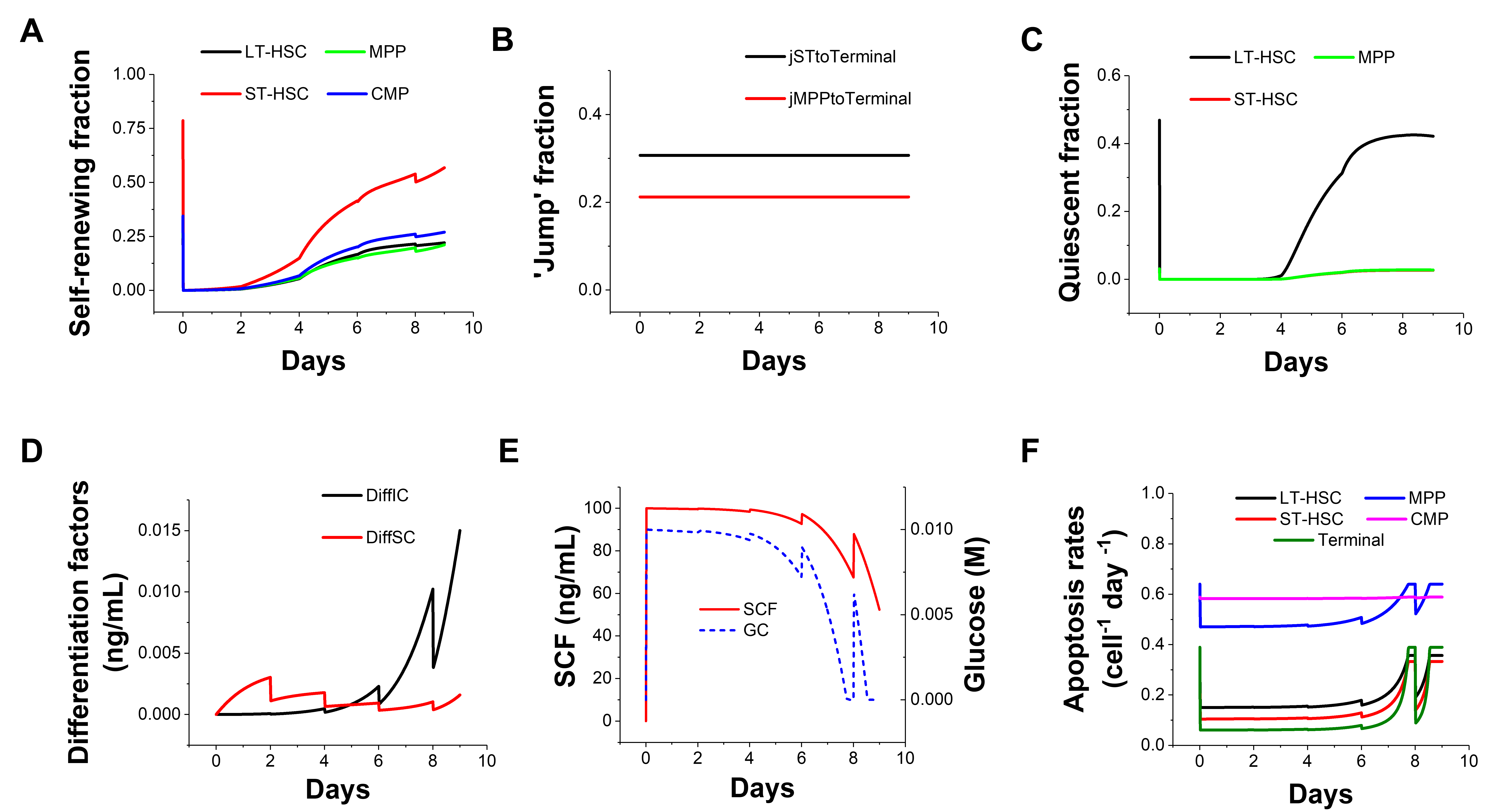

Supplement: S5 Fig — (A) self-renewing fractions of each sub-set rise steadily over time, also evidenced by the rising cell numbers. (B) fraction transitioning directly from ST-HSC or MPP to Terminal cells (jump). A higher number of ST-HSCs ‘jump’ to Terminal compared to MPPs, however each fraction is maintained over the entire culture period. (C) A higher fraction of LT-HSCs are predicted to remain quiescent while the more mature ST-HSC and MPP cells display a reduced quiescent capability. (D) The concentrations of differentiation stimulators and inhibitors in the culture media change dynamically over time, and is a function of cell-secreted cytokines. (E) Similar to the 3-state model, SCF and Glucose (GC) content is replenished every 2 days due to media exchange and, decreasing rapidly with increasing cell numbers. (F) Death (or apoptosis) rates of all cell types are predicted to remain constant while there is a high level of SCF and nutrient (Glucose availability) and increases with nutrient depletion. (TIFF) [file pone.0212502.s006.tiff]

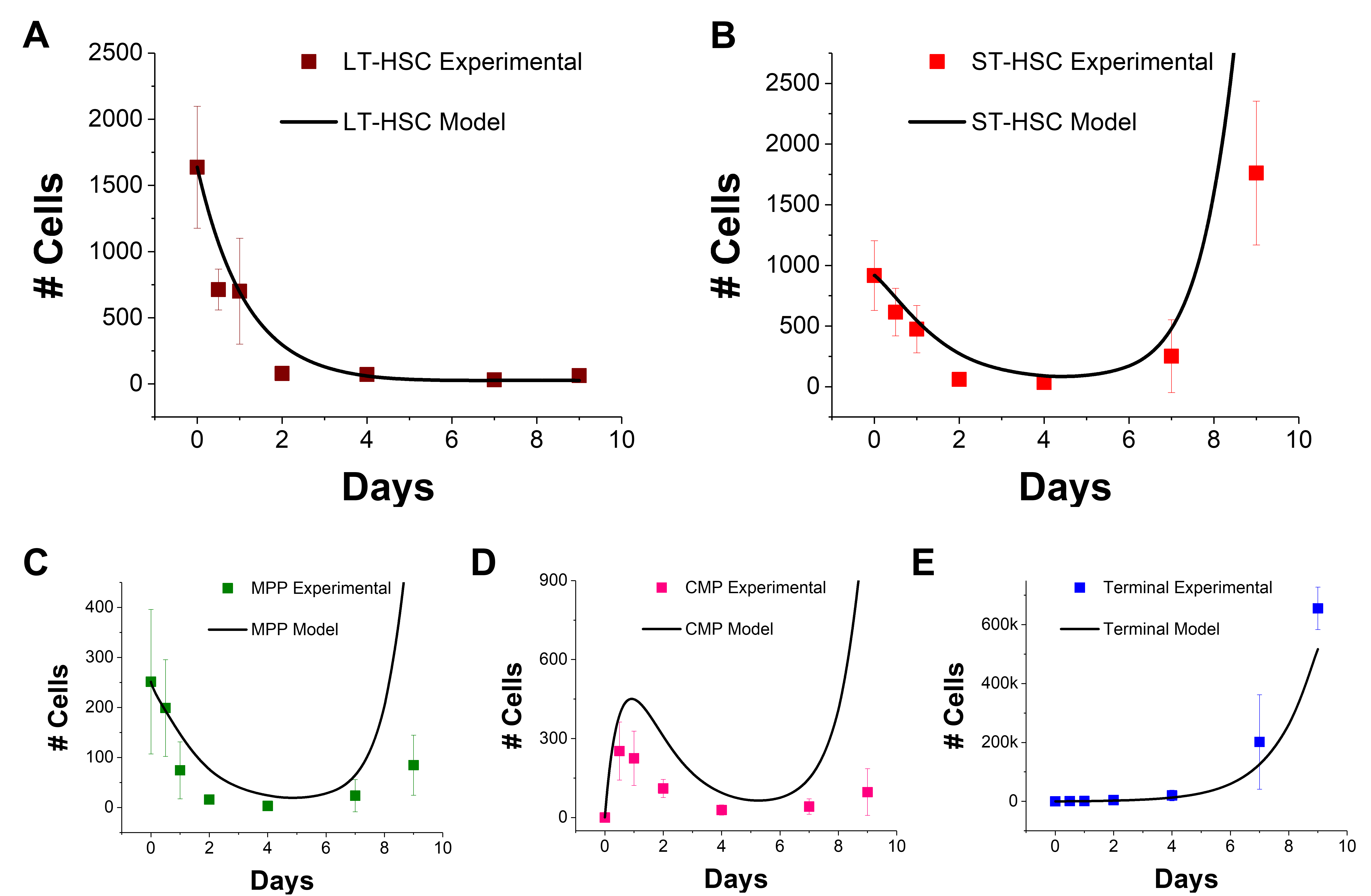

Supplement: S6 Fig — When the jSTHSCtoTerm and jMPPtoTerm values are set to 0, the model is unable to capture the experimental profiles for all cell populations. (A–E). The ST-HSC, MPP, and CMP populations exceed experimental observations, while Terminal cells are underpopulated due to lower initial differentiating cell numbers. (TIFF) [file pone.0212502.s007.tiff]

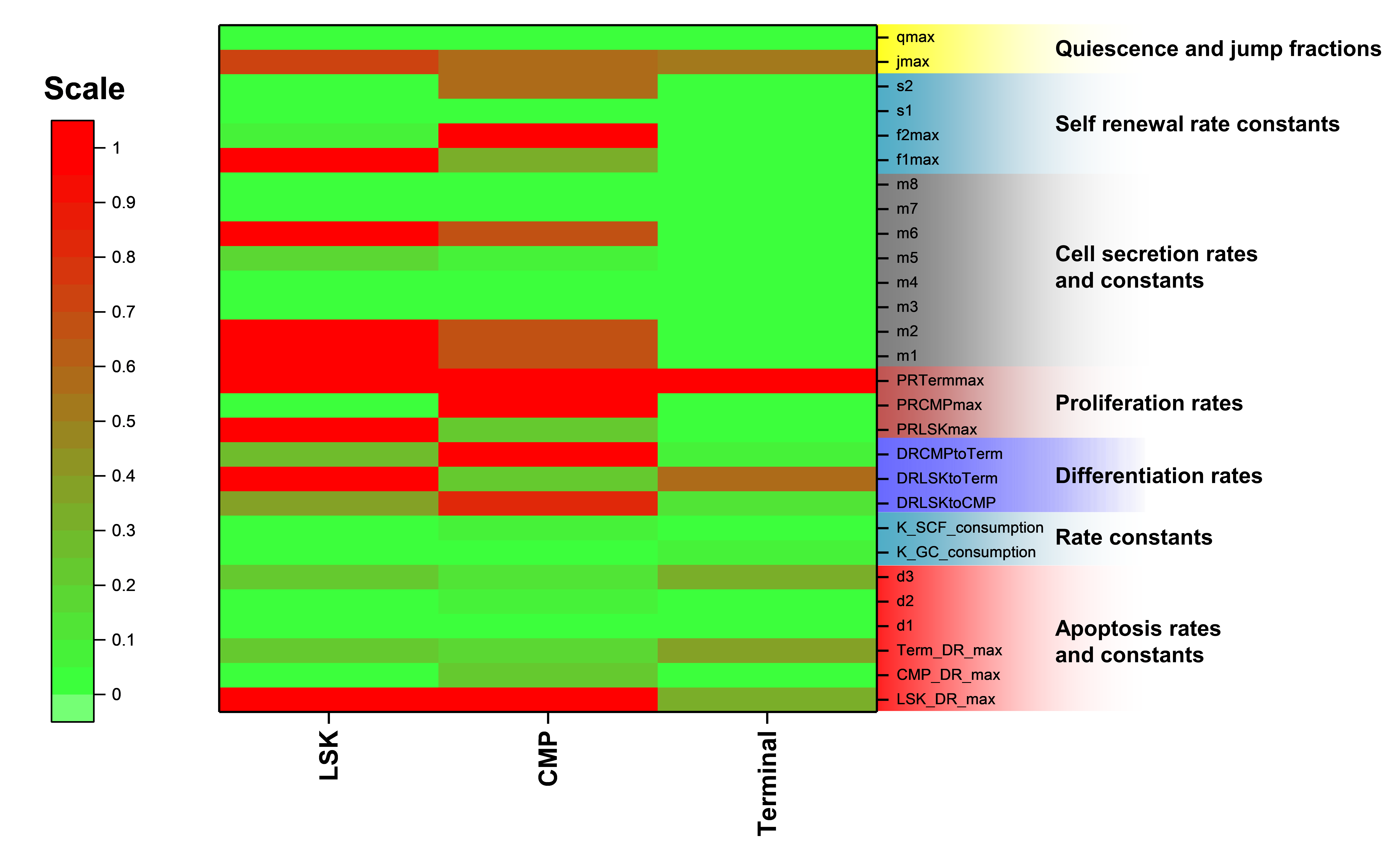

Supplement: S7 Fig — Red nodes in the matrix indicate that model sensitivity is >1% for a 1% change in parameter values. This is indicative of high parameter sensitivity and system instability, but also design parameters for future experimental optimization. Similar to the 5-state model, Terminal cells, on account of their large and heterogeneous populations are relatively insensitive to several model parameters except their proliferation rates (PRTermmax). (TIFF) [file pone.0212502.s008.tiff]

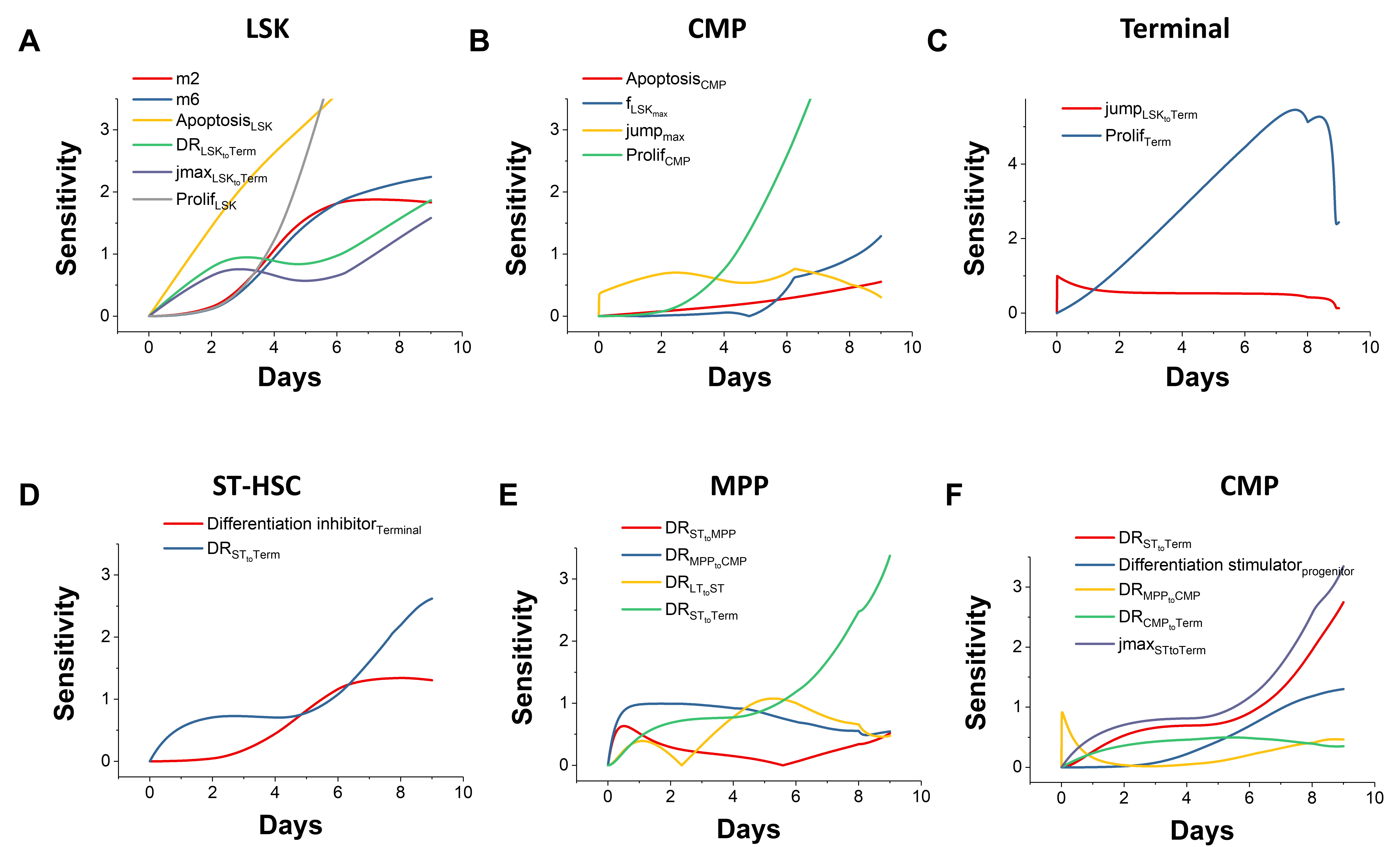

Supplement: S8 Fig — (A-C) For the 3 state model, changes in sensitivity for select parameters for each cell type indicate that the system response is highly non-linear. While the impact of some parameters steadily rises over time (ApoptosisLSK, ProlifCMP etc.), others plateau or decrease over time. (D-F). These dynamic profiles are also observed for the 5-state model. Cell state response to these factors can help us identify the positive or negative impact of specific parameters over time, and whether culture modulation can further help regulate system response. (TIFF) [file pone.0212502.s009.tiff]

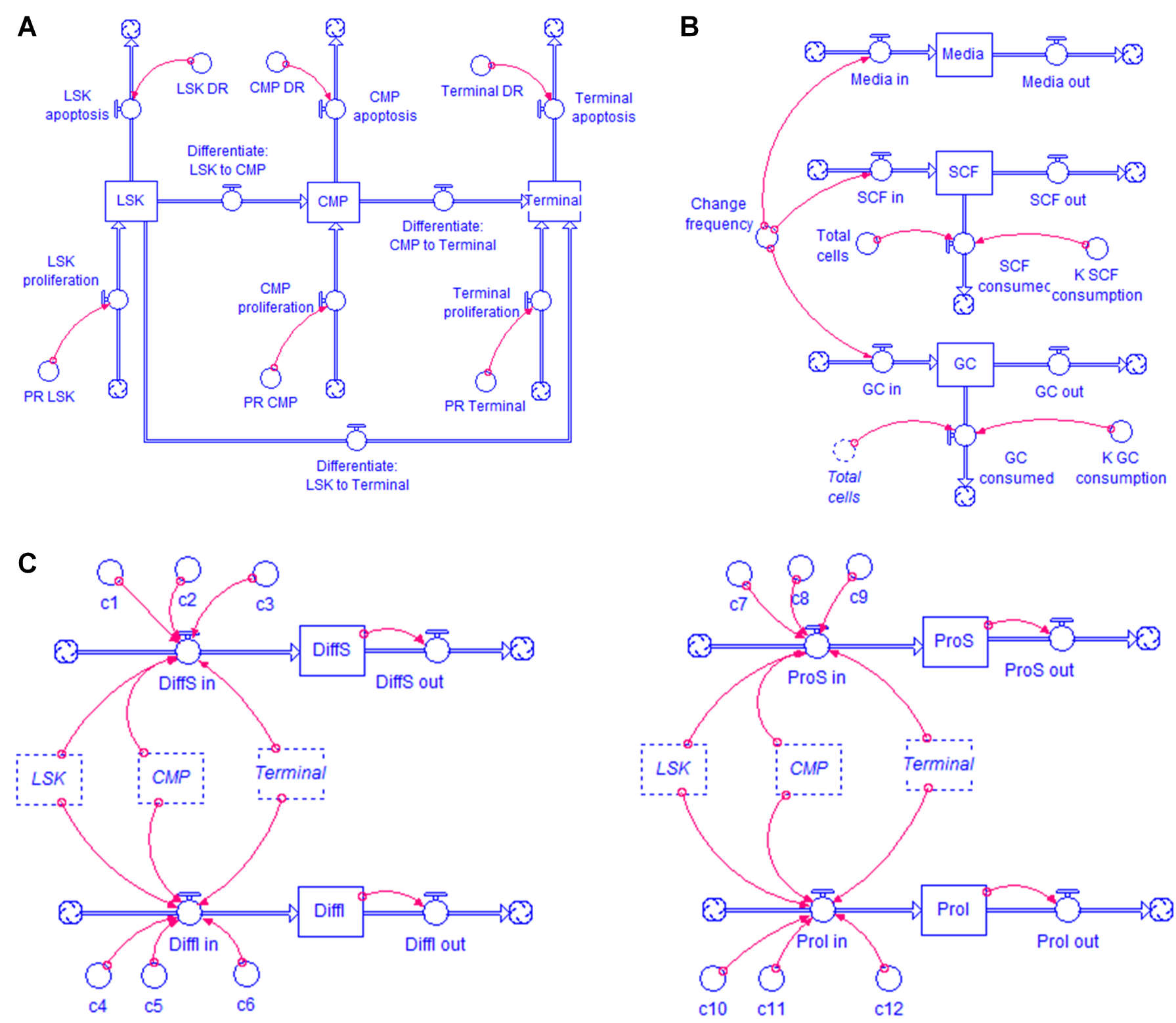

Supplement: S9 Fig — (A) Schematic of the overall differentiation process with input and output flows associated with each cell type. The inputs correspond to increase in cell population (proliferation, differentiation from previous state) whereas outputs correspond to decrease in cell population (apoptosis, differentiation into next state). Rates associated with each flow are described in the equations given in the Supplemental section. (B) Schematic of the 3 exogenous soluble components of the system: Media, SCF, nutrient availability (denoted as GC for Glucose). Exchanging the media replenishes both components and is controlled by the parameter ‘change frequency’. (C) Concentrations of groups of biomolecules (DiffS, DiffI, ProS, and ProI) are governed by the number of cells and a constant secretion rate associated with each cell type (c1 –c12) which dictate the self-renewing fractions (DiffS, DiffI) and the proliferation rates (ProS, ProI) for all cell types. (TIF) [file pone.0212502.s010.tif]

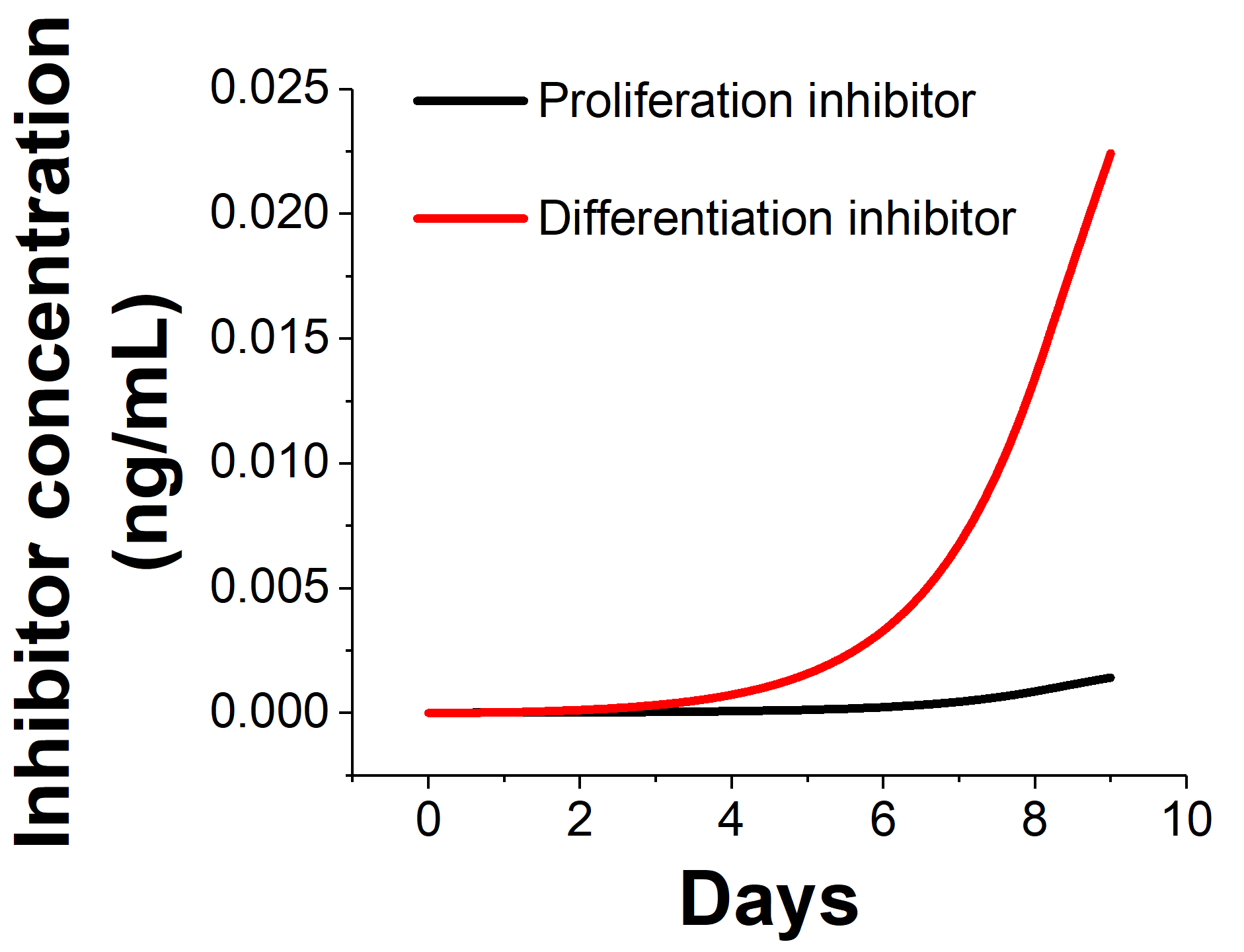

Supplement: S10 Fig — (TIFF) [file pone.0212502.s011.tiff]
